# Supplementary material for: Adipose stem cells are sexually dimorphic cells with dual roles as preadipocytes and resident fibroblasts
Source: Nat Commun. 2024 Sep 2;15:7643. doi: 10.1038/s41467-024-51867-9 (PMC11369120; doi:10.1038/s41467-024-51867-9)
Supplement: Supplementary file 1 — Supplementary information [file 41467_2024_51867_MOESM1_ESM.pdf]

Supplementary Information

for

Adipose stem cells are sexually dimorphic cells with dual roles as preadipocytes and resident fibroblasts

(Uhrbom et al.)

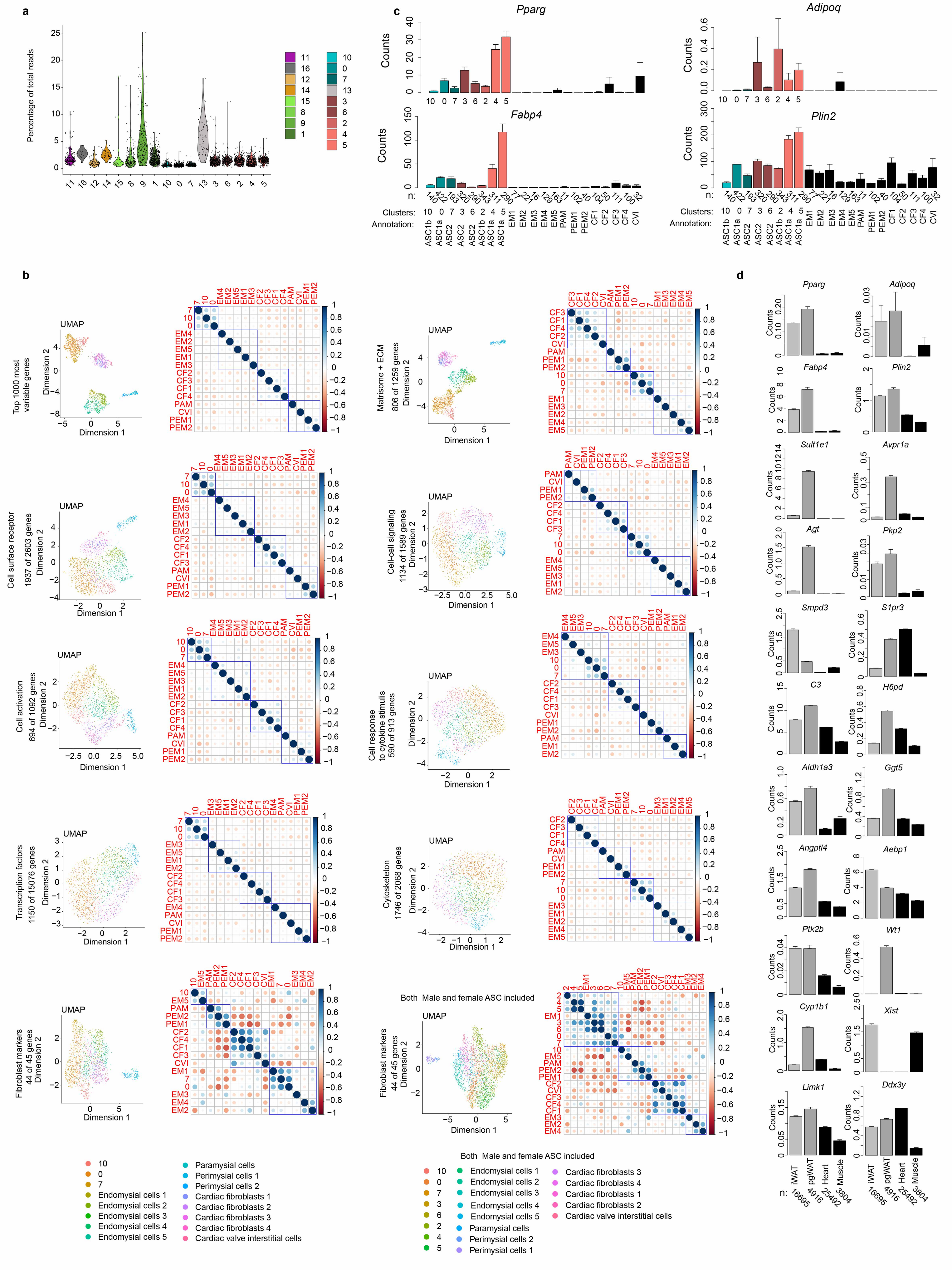

Supplementary figure 1 | Organotypic gene expression of ASC a. Percentage of total read counts derived from the 13 protein encoding genes from the mitochondrial genome. **b.** Comparison of fibroblasts in adipose (pgWAT), Skeletal muscle and heart using UMAP visualization and Pearson's r correlation plots with specific gene-sets as input. Blue lines encircle hierarchical clustered groups based on the Pearson's r value as in Figure 1c. Data from Skeletal muscle and heart are from previous published work[1] **c.** Barplots of common adipose genes *Pparg, Fabp4, Plin2* and *Adipoq* across fibroblast populations in adipose,heart and skeletal muscle. **d.** Barplots of the expression of the lipid metabolism associated genes enriched in adipose fibroblasts in previous published scRNA-seq datasets of fibroblasts[2] form iWAT[3,4], pgWAT[3,5], heart[6] and skeletal muscle[7,8]. Data are presented as mean values +/- SEM. n represent the number of cells. Abbreviations: ASC= adipose stem cells, CF1-4 = Cardiac fibroblasts 1-4,CVI = Cardiac valve interstitial cells, EM1-5 =Endomysial cells 1-5, i = inguinal, PAM = Paramysial cells, PEM1-2 = Perimysial cells 1-2, pg = perigonadal and WAT = White adipose tissue.

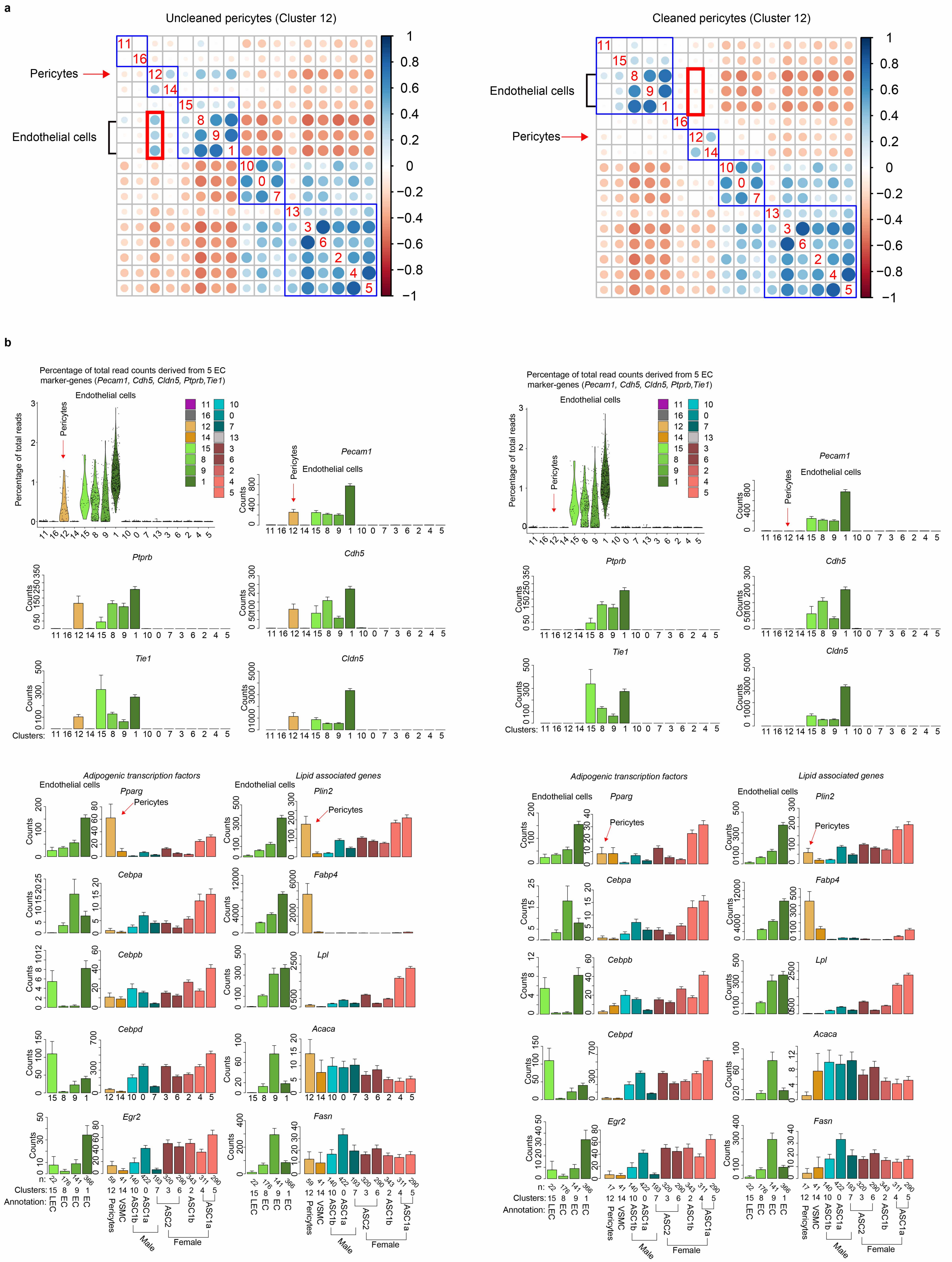

Supplementary figure 2 | Endothelial cell contamination of pericytes a. Hierarchical clustering as in Figure 1c with uncleaned pericytes (*Pecam1+,Cldn5+, Cdh5+, Tie1+, Ptprb+*;To the left) and with cleaned pericytes (*Pecam1-,Cldn5-, Cdh5-, Tie1-, Ptprb-*; To the right). **b**. Gene expression levels of adipogenic transcription factors and lipid associated genes in uncleaned (to the left) and cleaned (to the right) pericytes population. Data are presented as mean values +/- SEM. n represent number of cells. Abbreviations: ASC = Adipose stem cells, EC = Endothelial cells, LEC = Lymphatic EC, SEM=Standard error of mean and VSMC = Vascular smooth muscle cells.

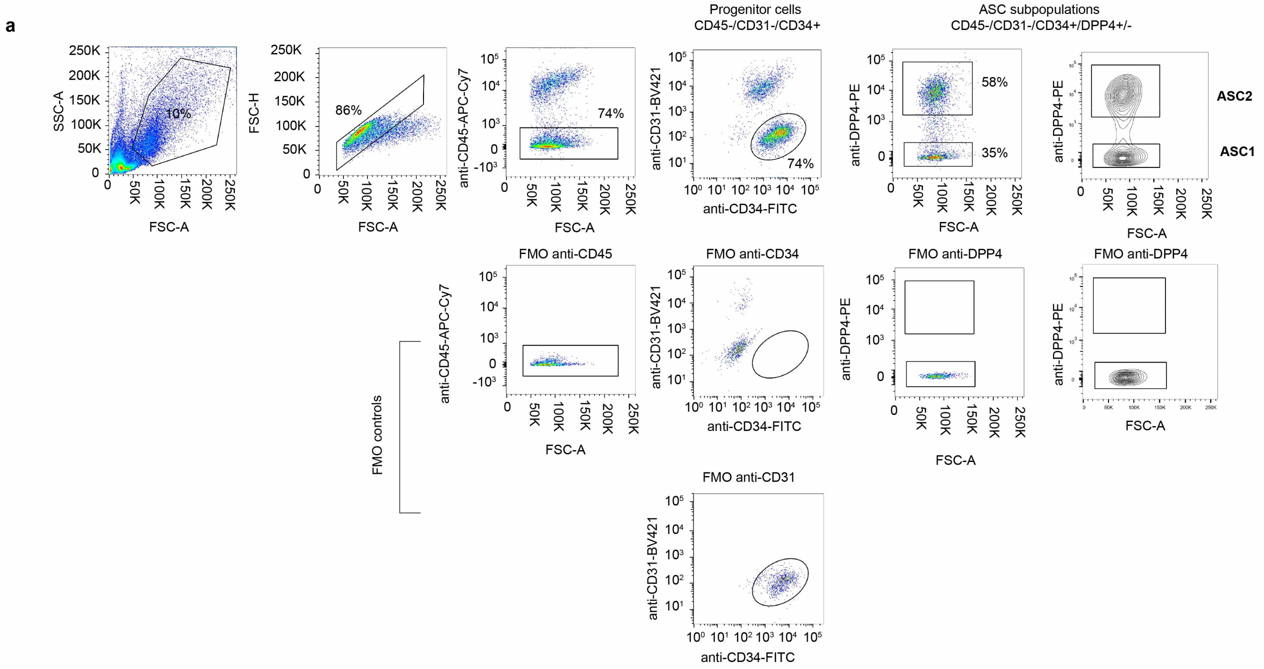

Supplementary figure 3 | FACS-sorting strategy for ASC subtype isolation a. FACS-sorting strategy for isolating ASC1 (DPP4-) and ASC2 (DPP4+) subpopulations from iWAT and pgWAT with fluorescence minus one controls. Figure shows iWAT and percentage number represent the proportion of cells in the gate. Abbreviations: ASC= Adipose stem cells, FACS = Fluorescence-activated cell sorting, FSC-H =Forward scatter-Height, i = inguinal, SSC-A = Side scatter-Area and WAT = White adipose tissue.

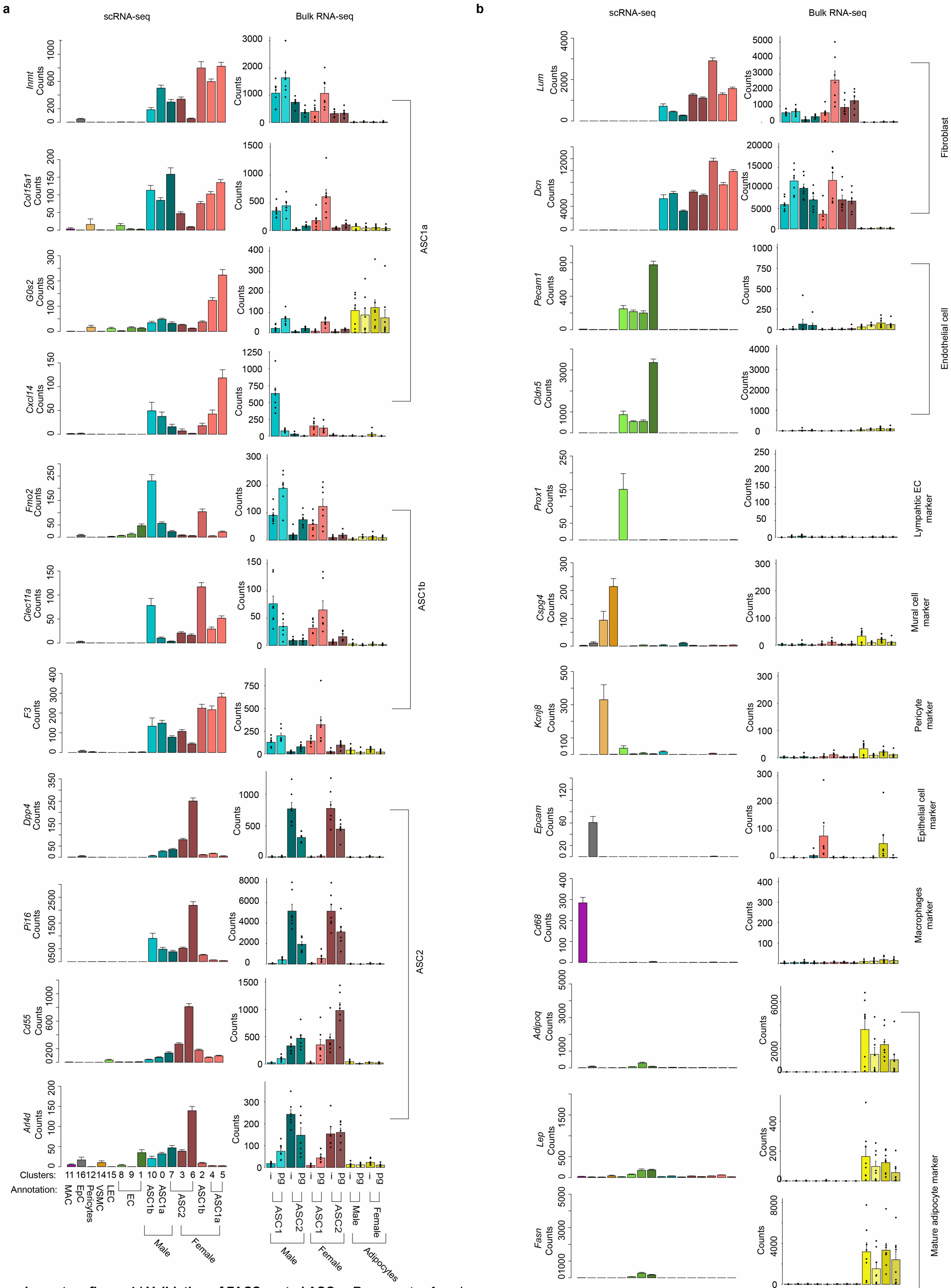

Supplementary figure 4 | Validation of FACS-sorted ASC a. Raw counts of marker genes for ASC1a, ASC1b and ASC2 cells in scRNA-seq and bulk RNA-seq of FACS sorted ASC subpopulations as well as bulk RNA-seq of mature adipocyte. **b.** same as in **a** but for cell type marker genes. Data are presented as mean values +/- SEM. For bulk RNA-seq samples n represent biological replicates. n=7 for all bulk samples except for ASC1 male iwat and mature adipocytes samples for which n=8. For scRNAseq data n represent number of cells, Cluster: 0 (n=422), 1(n=366), 2(n=343), 3(n=320), 4(n=311), 5(n=290), 6(n=229), 7(n=193), 8(n=176), 9(n=141), 10(n=140), 11(n=137), 12(n=17), 14(n=41), 15(n=22) and 16(n=19).Abbreviations: ASC= Adipose stem cells, EC= endothelial cells, EpC = Epithelial cells, FACS = Fluorescence-activated cell sorting, i = inguinal, LEC = Lymphatic EC, MAC= Macrophages, pg = perigonadal, sc =single cell, seq = sequencing, WAT = White adipose tissue and VSMC= Vascular smooth muscle cells.

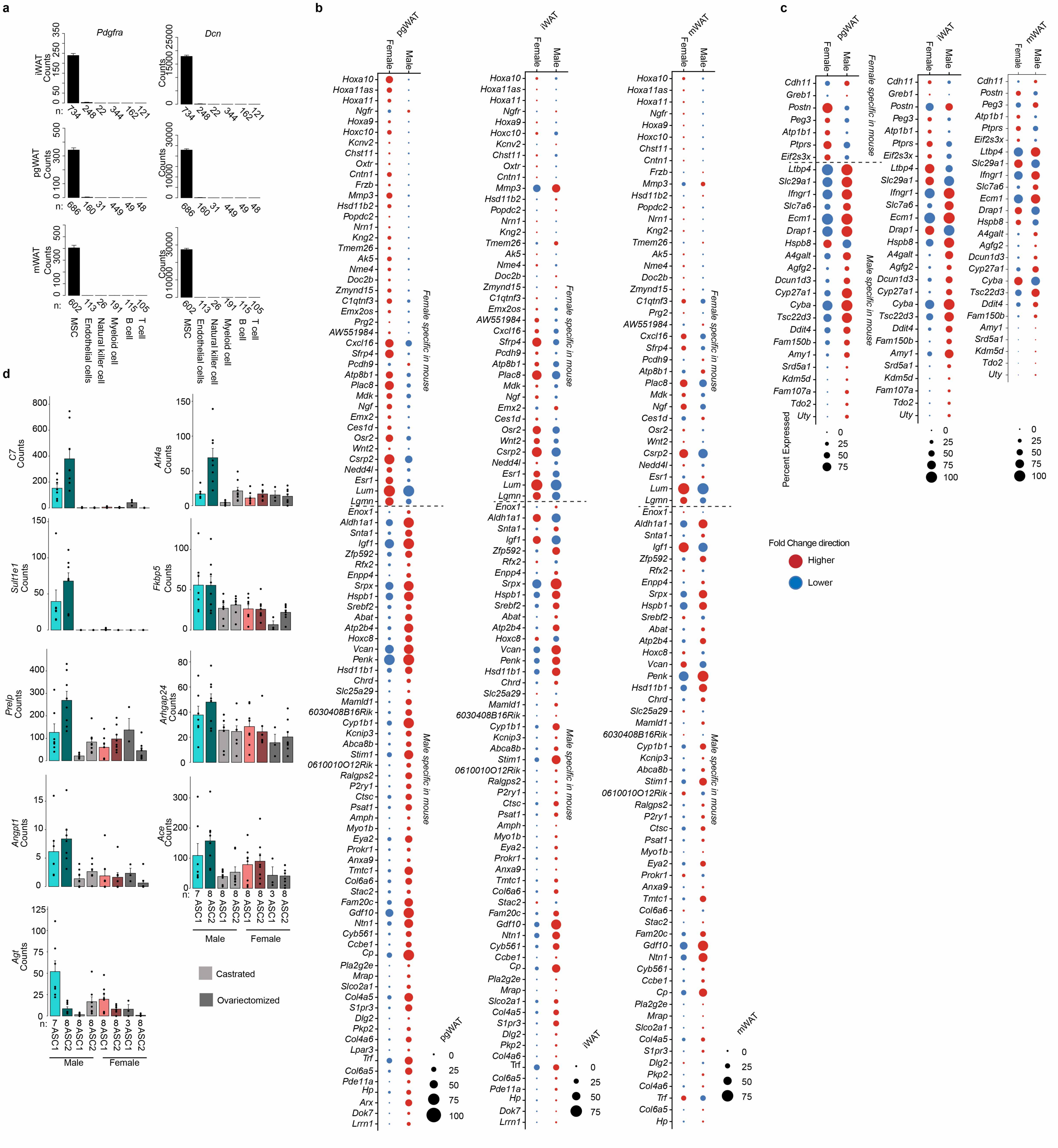

Supplementary figure 5 | Validation of the sexually dimorphic gene signature of ASC a. Expression of fibroblast marker genes *Pdgfra* and *Dcn* in identified cell types in iWAT, pgWAT and mWAT from Tabula Muris database[9]. n represent number of cells **b.** Dot plot of the expression of of the 104 sexual dimorphic genes in pgWAT ASC detected in MSC from iWAT, pgWAT and mWAT in the Tabula Muris database **c.** Same as in **c** but for the 29 DEGs specific for Bulk RNAseq samples. **d.** Barplots over the expression of 9 of the 36 sexually dimorphic genes impacted by castration of male mice, color code: control(colored bars) and castrated(grey bars)/ovariectomized (darkgrey bars) mice. *Sult1e1, C7, Prelp, Angpt1* and *Arl4a* were statistically significant different between male and female in the control samples, whereas they were not statistically significant different in the comparison between castrated male - female control group. The expression pattern of *Ace, Agt, Arhgap24* and *Fkbp5* indicates that their expression is driven by androgens, however the differences were not statistically significant in the control samples. n represent number of biological replicates. Abbreviations: ASC = Adipose stem cells, DEGs=Differentially expressed genes, FACS = Fluorescence-activated cell sorting, i = inguinal, MSC= Mesenchymal stem cells, m = mesenteric, pg = perigonadal and WAT= White adipose tissue.

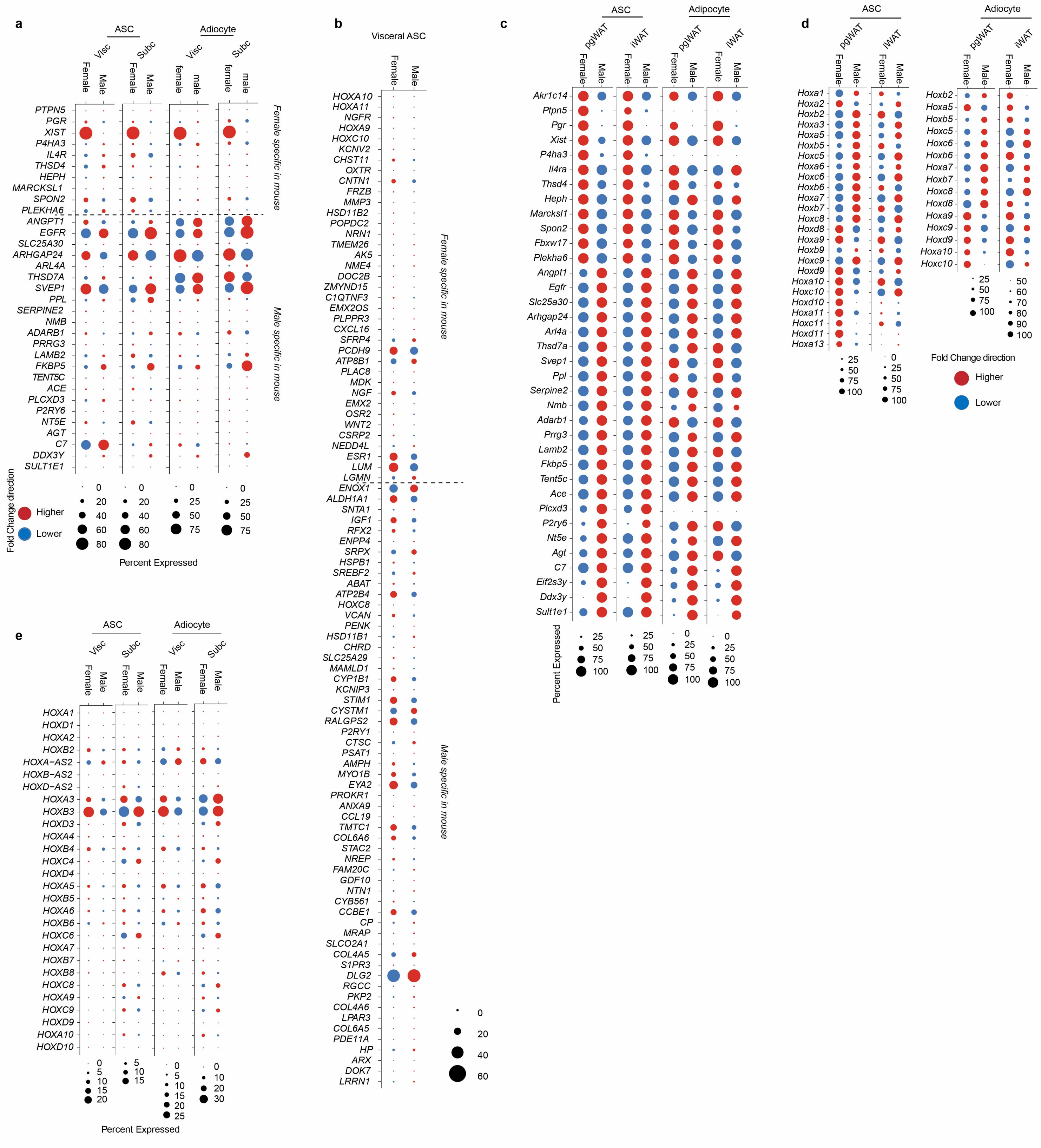

Supplementary figure 6 | Validation of the sexually dimorphic gene signature of ASC a. Dotplots showing the expression of 33 of the 36 sexual dimorphic genes in ASC and mature adipocytes from human adipose atlas database[10]. **b**. same as in **a** but for the 94 of the 104 sexual dimorphic DEGs in specific to pgWAT. **c.** Dotplots over the expression of the 36 sexual dimorphic genes in bulk RNA-seq samples of FACS sorted ASC and isolated adipocytes from iWAT and pgWAT. **d.** same as in **c** but for *hox* genes **e.** same as in **a** but for *hox* genes Abbreviations: ASC = Adipose stem cells, DEGs = Differentially expressed genes (DEGs), FACS = Fluorescence-activated cell sorting, i = inguinal, pg = perigonadal, Subc=subcutaneous, Visc = Visceral,WAT= White adipose tissue.

a Top 10 enriched Diseases and Biological functions from 36 sexual dimorphic genes

Categories	Diseases or Functions Annotation	p-value	Molecules	# Genes
Organismal Injury and Abnormalities,Reproductive System Disease	Damage of genital organ	7,43E-08	*Agt,Egfr,Il4ra,Sult1e1*	4
Metabolic Disease,Organismal Injury and Abnormalities	Glucose metabolism disorder	1,70E-07	*Ace,Adarb1,Agt,Akr1c14,C7,Ddx3y,Egfr,Eif2s3y,Fkbp5,Heph,Il4ra,Nt5e,P2ry6,Pgr,Serpine2,Sult1e1,Svep1,Xist*	18
Cardiovascular Disease,Organismal Injury and Abnormalities	Acute coronary syndrome	1,78E-07	*Ace,Agt,Egfr,Fkbp5,Nt5e,Pgr,Ptpn5*	7
Cardiovascular System Development and Function	Vascular resistance of kidney	5,48E-07	*Ace,Agt,Nt5e*	3
Developmental Disorder,Hereditary Disorder,Organismal Injury and Abnormalities,Renal and Urological Disease	Familial polycystic kidney disease	7,49E-07	*Ace,Agt,Angpt1,Egfr,Heph,Il4ra*	6
Endocrine System Disorders,Gastrointestinal Disease,Metabolic Disease,Organismal Injury and Abnormalities,Renal and Urological Disea	Early stage diabetic nephropathy	1,04E-06	*Ace,C7,Fkbp5,Nt5e,Xist*	5
Cancer,Cardiovascular Disease,Organismal Injury and Abnormalities,Skeletal and Muscular Disorders	Hyperplasia of vascular smooth muscle	1,13E-06	*Agt,Angpt1,Pgr*	3
Developmental Disorder,Hereditary Disorder,Organismal Injury and Abnormalities,Renal and Urological Disease	Autosomal dominant polycystic kidney disease	1,35E-06	*Ace,Agt,Angpt1,Heph,Il4ra*	5
Cardiovascular System Development and Function	Vasoconstriction of blood vessel	1,55E-06	*Ace,Agt,Egfr,P2ry6,Svep1*	5
Endocrine System Disorders,Metabolic Disease,Organismal Injury and Abnormalities	Impaired glucose tolerance	1,63E-06	*Ace,Agt,Egfr,Fkbp5,Il4ra,P2ry6*	6

b Genes associated with Glucose metabolism disorder

Gene	Fold Change log2	Prediction (based on measurement direction)	Findings	Measurement consistent with literature finding
Sult1e1	10,925	Affected	Affects (1)	Yes
Ddx3y	9,500	Affected	Affects (1)	Yes
Eif2s3y	8,389	Affected	Affects (1)	Yes
C7	6,502	Affected	Affects (1)	Yes
Agt	6,430	Increased	Increases (25)	Yes
Nt5e	5,021	Affected	Affects (7)	Yes
P2ry6	5,013	Decreased	Decreases (1)	Yes
Ace	3,365	Affected	Affects (90)	Yes
Fkbp5	2,846	Increased	Increases (4)	Yes
Adarb1	2,605	Increased	Increases (1)	Yes
Serpine2	2,370	Affected	Affects (1)	Yes
Svep1	2,101	Affected	Affects (2)	Yes
Egfr	1,521	Decreased	Decreases (9)	Yes
Heph	-2,375	Affected	Affects (1)	Yes
Il4ra	-3,542	Decreased	Increases (1)	No
Xist	-8,011	Affected	Affects (1)	Yes
Pgr	-10,779	Affected	Affects (15)	Yes
Akr1c14	-11,336	Affected	Affects (3)	Yes

“Prediction” column indicates if the measured fold direction increase, affect and decrease the function glucose metabolism disorder.
For example:
Increased: The gene is known to increase Glucose metabolism disorder and is upregulated in males, therefore predicted to increase the function.
Affected: The literature indicates this gene is involved in Glucose metabolism disorder but does not indicate whether it increases or decrease it.
Decreased: The gene is known to increase Glucose metabolism disorder and is downregulated in males, therefore predicted to decrease the function.
“Findings” column shows what the literature indicates if the expression of the gene is increased, affected or decreased during glucose metabolism disorder.
The number within the parentheses indicates the number of references supporting the finding.

c References for genes associated with Glucose metabolism disorder

Gene	Glucose metabolism disorder - References statment	Finding consistent with hypothesis
Sult1e1	Upregulation of human SULT1E1 mRNA in liver is associated with type 2 diabetes in female human.[11]	Yes
Agt (1)	Human ANGIOTENSIN II [product of AGT] protein increases insulin resistance in rat.[12]	Yes
Agt (2)	Upregulation of human AGT mRNA in carotid artery is associated with type 2 diabetes in human (population size: 21).[13]	Yes
Agt (3)	Numerous clinical trials with Angiotensinogen-II receptor blocker for the treatment for type-2-diabetes	Yes
C7	Upregulation of human C7 mRNA in mesangial cells from kidney is associated with early stage diabetic nephropathy in human.[14]	Yes
Ddx3y	Downregulation of mouse Ddx3y mRNA in pancreatic islets is associated with diabetes in mouse.[15]	No (Male specific finding
Eif2s3y	Downregulation of mouse Eif2s3y mRNA in pancreatic islets is associated with diabetes in mouse.[16]	No (Male specific finding)
Nt5e (1)	Upregulation of human NT5E mRNA in podocytes from kidney is associated with early stage diabetic nephropathy in human.[14]	Yes
Nt5e (2)	Upregulation of mouse Nt5e mRNA in islets of Langerhans is associated with type I diabetes in NOD mouse.[17]	Yes
Nt5e (3-7)	Downregulation of mouse CD73 [Nt5e] mRNA in vitro-differentiated induced Treg (iTreg) cells is associated with T1D [insulin-dependent diabetes mellitus] in NOD mouse.[18]	No
P2ry6 (1)	Mutation of mouse P2Y6 [P2ry6] gene to homozygous mutant mouse P2Y6 [P2ry6] gene (knockout) in skeletal muscle from Mus (mouse) increases impaired glucose homeostasis in Mus (mouse) that is increased by high fat diet.[19]	No
Ace (1)	Upregulation of mouse Ace mRNA in pancreatic cells is associated with hyperglycemia in female NOD mouse.[20]	Yes
Ace (2)	Homozygous mutant human ACE gene (insertion) decreases diabetic nephropathy in human exhibiting diabetes.[21]	Yes
Ace (3)	Upregulation of mouse Ace mRNA in pancreatic islets is associated with diabetes in mouse.[16]	Yes
Ace (4)	Upregulation of human ACE mRNA in carotid artery is associated with type 2 diabetes in human (population size: 21).[13]	Yes
Ace (5)	Downregulation of mouse Ace mRNA in mouse is associated with diabetic nephropathy in mouse.[22]	No
Ace (6)	Numerous clinical trials with ACEi for the treatment of diabetic nephropathy	Yes
Fkbp5 (1)	Mutation of mouse FKBP51 [Fkbp5] gene to mutant mouse FKBP51 [Fkbp5] gene (knockout) in mouse decreases insulin resistance in mouse that is increased by high fat diet.[23]	Yes
Fkbp5 (2)	Upregulation of mouse Fkbp5 mRNA in pancreatic islets is associated with diabetes in mouse.[16]	Yes
Fkbp5 (3)	Downregulation of human FKBP5 mRNA in podocytes from kidney is associated with early stage diabetic nephropathy in human.[14]	No
Fkbp5 (4)	Mutation of mouse FKBP51 [Fkbp5] gene to mutant mouse FKBP51 [Fkbp5] gene (knockout) in mouse decreases impaired glucose tolerance in mouse that involves high fat diet.[23]	Yes
Adarb1 (1)	In mouse, transgenic rat Adar2b [Adarb1] protein increases hyperglycemia in mouse.[24]	Yes
Serpine2 (1)	Downregulation of mouse Serpine2 mRNA in microglia from retina is associated with streptozotocin induced type1 diabetes in mouse.[25]	No
Svep1 (1)	Upregulation of mouse Svep1 mRNA in heart is associated with type 2 diabetes in Mus (mouse).[26]	Yes
Svep1 (2)	Upregulation of human SVEP1 mRNA is associated with diabetic nephropathy in Homo sapiens (human).[27]	Yes
Egfr(1)	In beta islet cells from 4 month-old male mouse, dominant negative transgenic mutant human EGFR protein (deletion) lacking kinase activity increases glucose intolerance in mouse.[28]	No
Egfr(2)	Upregulation of soluble mouse Egfr protein in serum is associated with streptozotocin-induced diabetes in male mouse.[29]	Yes
Egfr(3)	Upregulation of human EGFR protein in kidney is associated with diabetic nephropathy in human.[30]	Yes
Egfr(4-5)	Downregulation of soluble mouse Egfr protein in serum is associated with type 2 diabetes in male db/db mouse.[29]	No
Egfr(6-9)	Upregulation of mouse Egfr protein in sinusoidal endothelial cells from liver is associated with diabetes type 2 in mouse.[31]	Yes
Heph	Upregulation of human HEPH mRNA in placenta is associated with gestational diabetes mellitus in Homo sapiens (human).[32]	No (Female specific finding)
Xist	Downregulation of human XIST ncRNA in mesangial cells from kidney is associated with early stage diabetic nephropathy in human.[14]	Yes
Il4ra	Mutation of mouse Il4ra gene to homozygous mutant mouse Il4ra gene (knockout) in myeloid cells from Mus (mouse) decreases glucose intolerance in Mus (mouse) that is increased by high fat diet (20 weeks).[33]	No
Pgr(1-9)	Mifepristone, an inhibitor of human progesterone receptor [PGR] protein, has been approved as a drug for hyperglycemia associated with endogenous Cushing's syndrome in human exhibiting glucose intolerance.(FDA-Mifepristone)	Yes
Pgr(9-10)	Mifepristone, an inhibitor of human progesterone receptor [PGR] protein, is in Phase 4 clinical trial as a treatment for type 2 diabetes mellitus in human.(NCT05772169)	Yes
Pgr(11-15)	Medroxyprogesterone acetate, an activator of human PGR protein, is in Phase 3 clinical trial as a part of the combination drug estrogens, conjugated [conjugated estrogens/meprobamate] and progesterone to prevent diabetes mellitus in human. (NCT00000466)	No
Akr1c14 (1)	Downregulation of mouse Akr1c14 mRNA in mouse is associated with diabetic nephropathy in mouse.[22]	Yes
Akr1c14 (2-3)	Downregulation of mouse Akr1c14 mRNA in kidney is associated with diabetic kidney disease in db/db mouse.[30]	Yes

Supplementary figure 7 | Enriched Diseases and biological function a. The top 10 enriched diseases and biological functions from 36 sexual dimorphic genes, genes in the “Glucose metabolism disorder” term are colored in red. **b.**Table of genes associated with the term “Glucose metabolism disorder” and how the fold change in our data is related to previous findings. **c.** List of references for genes associated with “Glucose metabolism disorder” term and how the finding from the reference is consistent with the hypothesis that male ASC have a more severe metabolic glucose disorder than females. For example, if a knockout model of a male specific gene indicates protection against diabetes then the finding is consistent with the hypothesis that male have a more severe glucose metabolism disorder. On the other hand, if a knockout of a female specific gene decreases glucose intolerance (as for the gene *Il4ra*), then the finding is inconsistent with the hypothesis that male ASC have a more severe glucose metabolism disorder. Abbreviations: ASC = Adipose stem cells.

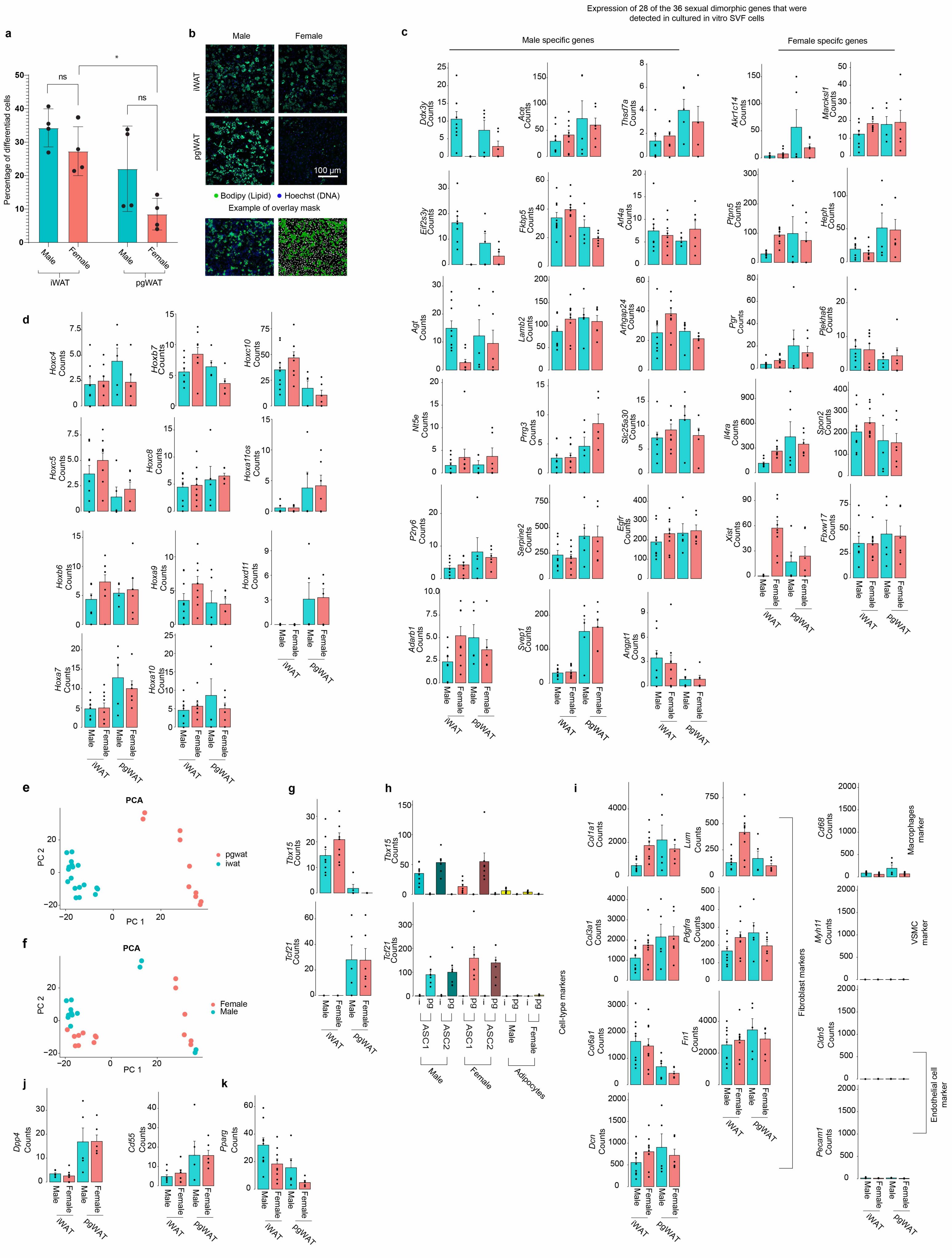

Supplementary figure 8 | In vitro differentiation of crude SVF cells from iWAT and pgWAT. a. Barplots over the level of differentiation of crude-SVF cells from iWAT and pgWAT from adult male and female mice, n = 4 four all groups. n represent biological replicates from independent experiments **b.** Representative images over differentiated of crude-SVF cells from iWAT and pgWAT from adult male and female mice, with example image on how the overlay mask method worked for identification of lipid filled cells. statistics were calculated with a two-way Anova, using Tukey's multiple comparison test (Prism). Adjusted P-values for multiple testing were used, *P-value=0.0317. **c**. Expression of 28 of the 36 detected genes in *in vitro* cultured crude SVF cells (Cell have been proliferated for four days and reached confluency prior to Bulk RNA-seq). Group size: Female pgWAT (n=6), male pgWAT (n=5), female iWAT (n=9) and male iWAT (n=9). n represent technical well replicates from two (pgWAT) and three (iWAT) independent experiments **d**. *Hox* gene expression in *in vitro* cultured crude SVF-cells **e**. PCA plots based on the top variables genes in cultured SVF cells from pgWAT and iWAT, blue color indicates iWAT and red color indicates pgWAT cells. **f**. Same as in **e** but blue color indicates male cells and red color indicates female cells **g**. Barplots showing the expression of iWAT (*Tbx15*) and pgWAT (*Tcf21*) specific transcription factors in cultured SVF cells. **h**. Same as in **g** but in freshly isolated FACS sorted ASC cells. n=7 for all bulk samples except for ASC1 male iwat and mature adipocytes samples for which n=8. n represent biological replicates **i**. Expression of cell-type markers in cultured SVF cells from iWAT and pgWAT. **j**. Expression of ASC2-markers in cultured SVF cells from iWAT and pgWAT. **k**. Expression of *Pparg* in cultured SVF cells from iWAT and pgWAT. Data are presented as mean values +/- SD for a and +/- SEM for all other barplots. Abbreviations: ASC = Adipose stem cells, EC= endothelial cells, EpC = Epithelial cells, ETC = Electron transport chain, FACS = Fluorescence-activated cell sorting, i = inguinal, LEC = Lymphatic EC, MAC= Macrophages,MSC= Mesenchymal stem cells, m = mesenteric, ns=not significant,PCA = Principal component analysis, pg = perigonadal, sc = single cell, SD=standard deviation, SEM=Standard error of the mean, seq=sequencing, SVF = Stromal vascular fraction, WAT= white adipose tissue and VSMC= Vascular smooth muscle cells.

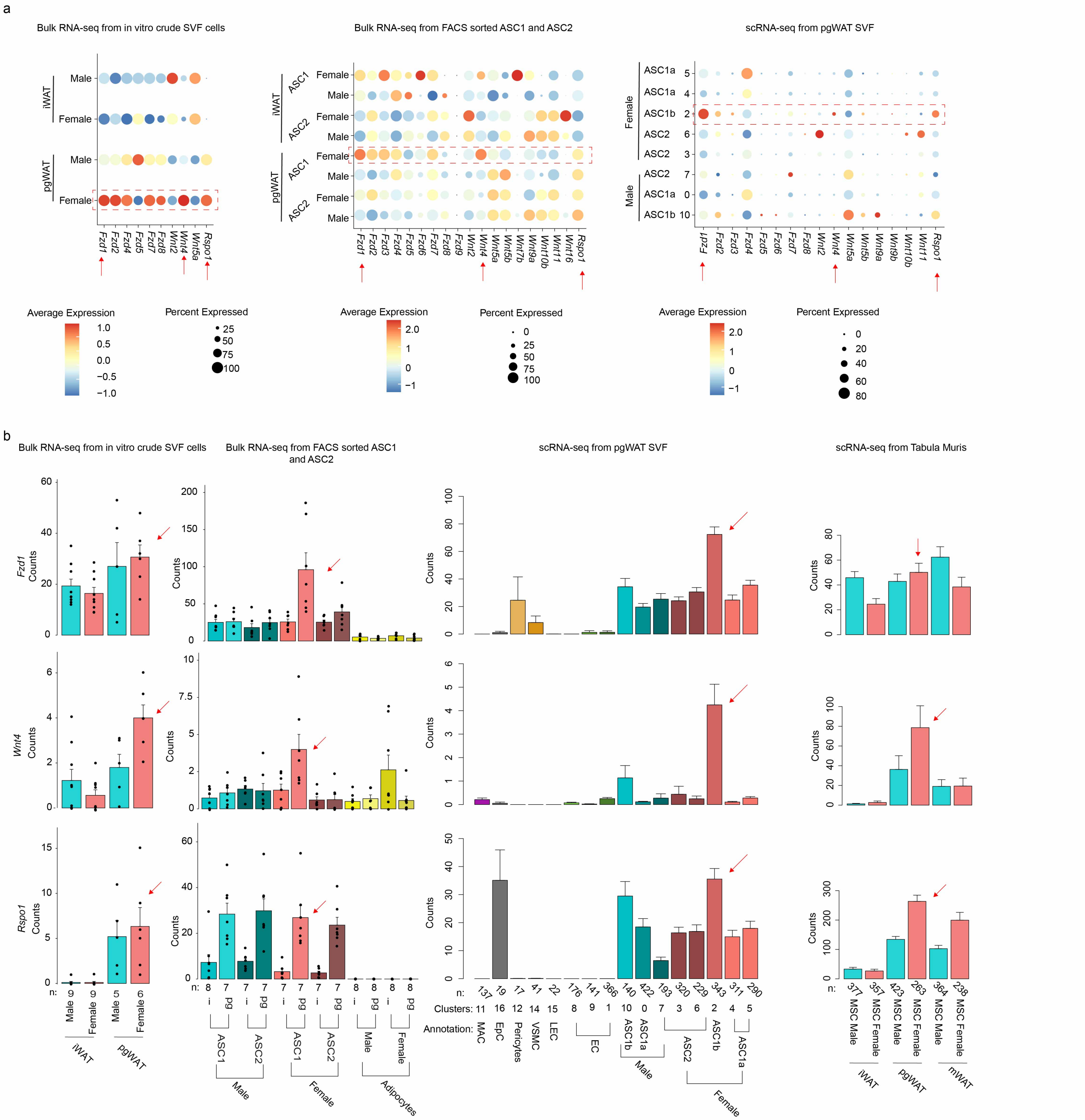

Supplementary figure 9 | Wnt signaling related transcripts in vitro and in vivo a. Dotplots over the scaled normalized expression in Bulk RNA-seq samples from *in vitro* cultured SVF cells, freshly isolated ASC1/2 cells and our scRNA-seq data from pgWAT.. Red lines indicates female samples from pgWAT. Red arrows highlight *Fzd1*, *Wnt4* and *Rspo1*. **b**. Barplots over the expression displaying the raw count reads of *Fzd1*, *Wnt4* and *Rspo1* in *in vitro* cultured SVF cells(n represent technical well replicates from two (pgWAT) and three (iWAT) independent experiments), freshly isolated ASC1/2 cells(n represent biological replicates), our and Tabula Muris scRNA-seq data[9](n represent number of cells). Abbreviations: ASC = Adipose stem cells, EC= endothelial cells, EpC = Epithelial cells, ETC = Electron transport chain, FACS = Fluorescence-activated cell sorting, i = inguinal, LEC = Lymphatic EC, MAC= Macrophages,MSC= Mesenchymal stem cells, m = mesenteric, ns=not significant,PCA = Principal component analysis, pg = perigonadal, sc = single cell, seq=sequencing, SVF = Stromal vascular fraction, WAT= white adipose tissue, Wnt = Wingless-related integration site and VSMC= Vascular smooth muscle cells.

Supplementary table 1: **Number of cells per cluster and mice in our scRNA-seq data.**

Cluster	0	1	2	3	4	5	6	7	8	9	10	11	12	13	14	15	16
**Cell number:**	422	366	343	320	311	290	229	193	176	141	140	137	59	52	41	22	19
**Male**	422	34	2	3	4	0	5	193	172	7	139	76	45	0	41	15	19
**Female**	0	332	341	317	307	290	224	0	4	134	1	61	14	52	0	7	0
**Mouse_1 (male)**	0	5	2	0	0	0	0	186	172	4	56	76	45	0	38	9	19
**Mouse_2 (female)**	0	162	137	155	119	197	139	0	3	58	0	0	11	0	0	0	0
**Mouse_3 (female)**	0	0	82	77	131	21	34	0	0	0	1	0	0	0	0	0	0
**Mouse_4 (male)**	295	0	0	0	0	0	0	1	0	0	67	0	0	0	1	0	0
**Mouse_5 (female)**	0	40	50	13	1	20	19	0	1	36	0	61	2	6	0	4	0
**Mouse_6_7 (females)**	0	130	72	72	56	52	32	0	0	40	0	0	1	46	0	3	0
**Mouse_8 (male)**	127	29	0	3	4	0	5	6	0	3	16	0	0	0	2	6	0

Supplementary table 2: **Number of cells per cluster from Lars Muhl et al[34].**

**Heart**	**Original Cluster#**	**Cells**	**Skeletal muscele**	**Original Cluster#**	**Cells**
Cardiac fibroblast 1	2	104	Endomysial cells 1	1	77
Cardiac fibroblast 2	3	50	Endomysial cells 2	3	221
Cardiac fibroblast 3	4	111	Endomysial cells 3	5	16
Cardiac fibroblast 4	5	100	Endomysial cells 4	6	129
Cardiac valve insterstitial cells	1	32	Endomysial cells 5	8	163
			Perimysial cells 1	2	102
			Perimysial cells 2	9	40
			Paramysial cells	4	11

Supplementary table 3: **Number of cells per tissue from Buechler et al[2].**

Tissue	Cell number	Reference PMID	Author	Sex	Mouse background	10x chemistry
pgWAT Fibroblasts	4916	30265241 / 29937373	Hepler, Strand[xx] / Burl, Granneman[xx]	M /M	C57BL/6 (MuralChaser mice (Hepler))	v2 / v2
iWAT Fibroblasts	16695	29937373 / 29925944	Burl, Granneman[xx]/ Schwalie, Deplancke[xx]	M / M&F	C57BL/6 (Dlk -RFP (Schwaile))	v2 / v2
Muscle Fibroblasts	3804	30890574 / 31809738	DellOrso, Sartorelli[xx]/ Scott, Underhill[xx]	? / M & F	C57BL/6 (Hic1CreERT2; Rosa26LSL - tdTomato (Scott))	v2 / v2
Heart Fibroblasts	25492	32142665	Soilman, Rossi[xx]	M & F	C57BL/6 (PdgfraH2BeGFP and Hic1 - citrine)	v2 / v2

Supplementary table 4: **Chromosome location and gene biotype of the 36 sexual dimorphic genes in ASC.**

Gene name	Ensembl_gene_id	Entrez Gene Name	Sex	Fold change Log2	Location	Type(s)	Chromsome location
Akr1c14	ENSMUSG00000033715	aldo-keto reductase family 1, member C14	Female	-11,3	Cytoplasm	enzyme	13
Ptpn5	ENSMUSG00000030854	protein tyrosine phosphatase non-receptor type 5	Female	-10,9	Plasma Membrane	phosphatase	7
Pgr	ENSMUSG00000031870	progesterone receptor	Female	-10,8	Nucleus	ligand-dependent nuclear receptor	9
Xist	ENSMUSG00000086503	X inactive specific transcript	Female	-8,0	Nucleus	other	X
P4ha3	ENSMUSG00000051048	prolyl 4-hydroxylase subunit alpha 3	Female	-6,6	Cytoplasm	enzyme	7
Il4ra	ENSMUSG00000030748	interleukin 4 receptor	Female	-3,5	Plasma Membrane	transmembrane receptor	7
Thsd4	ENSMUSG00000032289	thrombospondin type 1 domain containing 4	Female	-3,4	Cytoplasm	other	9
Heph	ENSMUSG00000031209	hephaestin	Female	-2,4	Plasma Membrane	transporter	X
Marcksl1	ENSMUSG00000047945	MARCKS like 1	Female	-2,2	Cytoplasm	other	4
Spon2	ENSMUSG00000037379	spondin 2	Female	-1,9	Extracellular Space	other	5
Fbxw17	ENSMUSG00000037816	F-box and WD-40 domain protein 17	Female	-1,7	Other	other	13
Plekha6	ENSMUSG00000041757	pleckstrin homology domain containing, family A member 6	Female	-1,5	Other	other	1
Angpt1	ENSMUSG00000022309	angiopoietin 1	Male	1,4	Extracellular Space	growth factor	15
Egfr	ENSMUSG00000020122	epidermal growth factor receptor	Male	1,5	Plasma Membrane	kinase	11
Slc25a30	ENSMUSG00000022003	solute carrier family 25 member 30	Male	1,6	Cytoplasm	transporter	14
Arhgap24	ENSMUSG00000057315	Rho GTPase activating protein 24	Male	1,7	Cytoplasm	other	5
Arl4a	ENSMUSG00000047446	ADP ribosylation factor like GTPase 4A	Male	1,9	Nucleus	enzyme	12
Thsd7a	ENSMUSG00000032625	thrombospondin type 1 domain containing 7A	Male	2,1	Cytoplasm	other	6
Svep1	ENSMUSG00000028369	sushi, von Willebrand factor type A, EGF and pentraxin domain containing 1	Male	2,1	Cytoplasm	other	4
Ppl	ENSMUSG00000039457	periplakin	Male	2,2	Cytoplasm	other	16
Serpine2	ENSMUSG00000026249	serpin family E member 2	Male	2,4	Extracellular Space	other	1
Nmb	ENSMUSG00000025723	neuromedin B	Male	2,5	Extracellular Space	other	7
Adarb1	ENSMUSG00000020262	adenosine deaminase RNA specific B1	Male	2,6	Nucleus	enzyme	10
Prrg3	ENSMUSG00000033361	proline rich and Gla domain 3	Male	2,6	Plasma Membrane	other	X
Lamb2	ENSMUSG00000052911	laminin subunit beta 2	Male	2,7	Extracellular Space	enzyme	9
Fkbp5	ENSMUSG00000024222	FKBP prolyl isomerase 5	Male	2,8	Nucleus	enzyme	17
Tent5c	ENSMUSG00000044468	terminal nucleotidyltransferase 5C	Male	3,0	Extracellular Space	enzyme	3
Ace	ENSMUSG00000020681	angiotensin I converting enzyme	Male	3,4	Plasma Membrane	peptidase	11
Plcxd3	ENSMUSG00000049148	phosphatidylinositol specific phospholipase C X domain containing 3	Male	3,9	Plasma Membrane	other	15
P2ry6	ENSMUSG00000048779	pyrimidinergic receptor P2Y6	Male	5,0	Plasma Membrane	G-protein coupled receptor	7
Nt5e	ENSMUSG00000032420	5'-nucleotidase ecto	Male	5,0	Plasma Membrane	phosphatase	9
Agt	ENSMUSG00000031980	angiotensinogen	Male	6,4	Extracellular Space	growth factor	8
C7	ENSMUSG00000079105	complement C7	Male	6,5	Extracellular Space	other	15
Eif2s3y	ENSMUSG00000069049	eukaryotic translation initiation factor 2, subunit 3, structural gene Y-linked	Male	8,4	Cytoplasm	translation regulator	Y
Ddx3y	ENSMUSG00000069045	DEAD-box helicase 3 Y-linked	Male	9,5	Cytoplasm	enzyme	Y
Sult1e1	ENSMUSG00000029272	sulfotransferase family 1E member 1	Male	10,9	Cytoplasm	enzyme	5

Supplementary table 5: **Number of cells per depot and sex from Tabula Muris[9].**

Depot /sex	MSC of adipose	Myeloid cell	Endothelial cell	Natural killer cells	B cell	T Cell
**pgWAT**	686	449	160	31	49	48
Male	423	252	132	12	22	28
Female	263	197	28	19	27	20
**iWAT**	734	344	248	22	162	121
Male	377	159	142	14	107	86
Female	357	185	106	8	55	35
**mWAT**	602	191	113	26	115	105
Male	364	53	77	5	71	65
Female	238	138	36	21	44	40

Supplementary table 6: **Number of cells per cell type and depot from Emont et al[10].**

Depot	Cells	Male	Female
Subcutaneous ASPC	16880	5852	11028
Visceral ASPC	14646	5334	9312
Subcutaneous Mature adipocytes	14396	3610	10786
Visceral Mature adipocytes	11475	3322	8153

Supplementary table 7: **Antibodies used for imaging**

Antigen	Gene	Supplier	Cat#	host	Clone	Lot	Dilution used	Label
CD31	*Pecam1*	R&D Systems	AF3628	goat		YZU0114021 / YZU0121071	1:200- 1:300	
CD31	*Pecam1*	BD Bioscience	550274	rat	MEC13.3	53198	1:100	
CD31	*Pecam1*	Abcam	ab28364	rabbit		GR324774211	1:50	
DPP4(CD26)	*Dpp4*	R&D Systems	AF954	goat		GJT0318011	1:100- 1:200	
PDGFRalpha	*Pdgfra*	R&D Systems	AF1062	goat		HMQ0216021	1:100- 1:200	
NGFR	*Ngfr*	Abcam	Ab52987	rabbit		GR32384033	1:100	
goat IgG	*	Invitrogen	A21082	donkey		1889311 / 2309146	1:500	AlexaFluor-633 conjugated
goat IgG	*	Invitrogen	A21432	donkey		1818686	1:500	AlexaFluor-555 conjugated
rabbit IgG	*	Invitrogen	A10043	donkey		1917929 / 2165747	1:500	AlexaFluor-680 conjugated
rabbit IgG	*	Invitrogen	A31572	donkey		1837922	1:500	AlexaFluor-555 conjugated
rat IgG	*	Invitrogen	A21208	donkey		1900239	1:500	AlexaFluor-488 conjugated
rat IgG	*	Invitrogen	SA5-10029	donkey		RK2304679	1:500	DyLight-650 conjugated
rat IgG	*	Invitrogen	SA5-10030	donkey		VC2968071	1:200	DyLight-680 conjugated
rat IgG	*	Jackson Laboratories	712-165-153	donkey		139289	1:300	Cy3-conjugated
*Secondary Antibody								

Supplementary table 8: **Antibodies used for Fluorescent activated cell sorting (FACS)**

Antigen	Gene	Supplier	Cat#	Reactivity	Isotype	Primary/Secondary Mono-/Polyclonal	Stock Concentration	Dilution Used	Label
CD31	*Pecam1*	BD Bioscience	562939	Mouse	RAT LEW IgG2a K	Primary-Monoclonal	0.2 mg/ml	1:25	BV421
CD45	*Ptprc*	BD Bioscience	557659	Mouse	RAT (LOU) IgG2bK	Primary-Monoclonal	0.2 mg/ml	1:25	APC-Cy7
CD34	*Cd34*	BD Bioscience	553733	Mouse	Rat IgG2a, κ	Primary-Monoclonal	0.5 mg/ml	1:25	FITC
CD26 (DPP4)	*Dpp4*	Biolegend	137804	Mouse	Rat IgG2a, k	Primary-Monoclonal	0.2 mg/ml	1:150	PE

Supplementary table 9: **Medium and enzymes used for digestion of tissues**

Enzymes	Supplier	Identifier (Cat#)
Collagenase I	ThermoFisher Scientific	17100017
Collagenase II	Worthington	4176
Dispase II	Sigma -Aldrich	D4693
DNAse	ThermoFisher Scientific	90083
Collagenase IV	Sigma -Aldrich	C4 -BIOC
**Medium/kits**		
DMEM	ThermoFisher Scientific	21885 -025
Skeletal Muscle Dissociation kit	Miltenyi Biotec	130 -098 -305

Supplementary table 10: **Key reagents for ASC proliferation, differentiation and imaging**

Medium	Supplier	Identifier (Cat#)
Subcutaneous Preadipocyte medium	Zenbio	PM-1
Subcutaneous Basal Medium	Zenbio	BM -1
Other reagents		
Basic FGF	Sigma-Aldrich	F0291
IBMX	Sigma-Aldrich	I5879
Dexamethasone	Sigma-Aldrich	D2915
Insulin, human recombinant	Novo Nordisk	
Pioglitazone	AstraZeneca	N/A
Angiotensin II	Sigma-Aldrich	A9525 -5X1MG
Cell staining		
Hoechst 33342	ThermoFisher Scientific	H3570
Bodipy	ThermoFisher Scientific	D3922
Propidium Iodine	ThermoFisher Scientific	P3566

Supplementary table 11: **Primers used for qPCR (Method: SYBR Green)**

Gene	Full name	Fwd	Rev	Source	Primer bank ID
Adipoq	Adiponectin	GCACTGGCAAGTTCTACTGCAA	GTAGGTGAAGAGAACGGCCTTGT	White Adipocytes Cultured under Membranes Maintain Identity, Function, and Can Transdifferentiate into Brown-like Adipocytes,	N/A
DPP4	Dipeptidyl peptidase 4	CCGTGGAAGGTTCTTCTGGG	GCTGCCGCTTCATCTTTGC	Athanasia Spandidos, Xiaowei Wang, Huajun Wang and Brian Seed: PrimerBank: a resource of human and mouse PCR primer pairs for gene expression detection and quantification. Nucl. Acids Res. 2010 38: D792-9.	227116290c3
Fabp4	Fatty acid binding protein 4	ACACCGAGATTTCCTTCAAACTG	CCATCTAGGGTTATGATGCTCTTCA	Harms et al. Prdm16 is required for the maintenance of Brown Adipocyte Identity and function in adult mice. Cell Metab, Volume 19, Issue 4, 1 April 2014, Pages 593-604	N/A
Lep	Leptin	TGTGGCTTTGGTCCTATCTGT	GTGAAGCCCAGGAATGAAGTC	Self-designed primers	N/A
LPL	Lipoprotein lipase	AAGACCTTCGTGGTGATCCAT	GCCAGCTGACACTGGATAATG	Harms et al. Mature Human White Adipocytes Cultured under Membranes Maintain Identity, Function, and Can Transdifferentiate into Brown-like Adipocytes, Volume 27, Issue 1, 2 April 2019, Pages 213-225.e5	N/A
PPARg2	Peroxisome proliferator-activated receptor gamma 2	TGGCATCTCTGTGTCAACCATG	GCATGGTGCCTTCGCTGA	Harms et al. Prdm16 is required for the maintenance of Brown Adipocyte Identity and function in adult mice. Cell Metab, Volume 19, Issue 4, 1 April 2014, Pages 593-604	N/A
TBP	TATA-binding protein	GAAGCTGCGGTACAATTCCAG	CCCCTTGTACCCTTCACCAAT	Harms et al. Prdm16 is required for the maintenance of Brown Adipocyte Identity and function in adult mice. Cell Metab, Volume 19, Issue 4, 1 April 2014, Pages 593-604	N/A

Supplementary table 12: **Settings for multi-wavelength cell scoring**

Dye	Hoechst	Bodipy	Propidium Iodine
Stained area	Nucleai	Cytoplasm	Nucleai
Approximate min width	8	8	8
Approximate Max width	14	16	14
Intensity above local background	2000	8000	5000
Minimum stained area	30	30	30

References

1. Muhl L, Genove G, Leptidis S, Liu J, He L, Mocci G, et al. Single-cell analysis uncovers fibroblast heterogeneity and criteria for fibroblast and mural cell identification and discrimination. Nat Commun **11**, 3953 (2020).

2. Buechler MB, Pradhan RN, Krishnamurty AT, Cox C, Calviello AK, Wang AW, et al. Cross-tissue organization of the fibroblast lineage. Nature **593**, 575-9 (2021).

3. Burl RB, Ramseyer VD, Rondini EA, Pique-Regi R, Lee YH, Granneman JG. Deconstructing Adipogenesis Induced by beta3-Adrenergic Receptor Activation with Single-Cell Expression Profiling. Cell Metab **28**, 300-9 e4 (2018).

4. Schwalie PC, Dong H, Zachara M, Russeil J, Alpern D, Akchiche N, et al. A stromal cell population that inhibits adipogenesis in mammalian fat depots. Nature **559**, 103-8 (2018).

5. Hepler C, Shan B, Zhang Q, Henry GH, Shao M, Vishvanath L, et al. Identification of functionally distinct fibro-inflammatory and adipogenic stromal subpopulations in visceral adipose tissue of adult mice. Elife **7**, (2018).

6. Soliman H, Paylor B, Scott RW, Lemos DR, Chang C, Arostegui M, et al. Pathogenic Potential of Hic1-Expressing Cardiac Stromal Progenitors. Cell Stem Cell **26**, 459-61 (2020).

7. Dell'Orso S, Juan AH, Ko KD, Naz F, Perovanovic J, Gutierrez-Cruz G, et al. Single cell analysis of adult mouse skeletal muscle stem cells in homeostatic and regenerative conditions. Development **146**, (2019).

8. Scott RW, Arostegui M, Schweitzer R, Rossi FMV, Underhill TM. Hic1 Defines Quiescent Mesenchymal Progenitor Subpopulations with Distinct Functions and Fates in Skeletal Muscle Regeneration. Cell Stem Cell **25**, 797-813 e9 (2019).

9. Tabula Muris C, Overall c, Logistical c, Organ c, processing, Library p, et al. Single-cell transcriptomics of 20 mouse organs creates a Tabula Muris. Nature **562**, 367-72 (2018).

10. Emont MP, Jacobs C, Essene AL, Pant D, Tenen D, Colleluori G, et al. A single-cell atlas of human and mouse white adipose tissue. Nature **603**, 926-33 (2022).

11. Fashe M, Yi M, Sueyoshi T, Negishi M. Sex-specific expression mechanism of hepatic estrogen inactivating enzyme and transporters in diabetic women. Biochem Pharmacol **190**, 114662 (2021).

12. Ogihara T, Asano T, Ando K, Chiba Y, Sakoda H, Anai M, et al. Angiotensin II-induced insulin resistance is associated with enhanced insulin signaling. Hypertension **40**, 872-9 (2002).

13. Hodroj W, Legedz L, Foudi N, Cerutti C, Bourdillon MC, Feugier P, et al. Increased insulin-stimulated expression of arterial angiotensinogen and angiotensin type 1 receptor in patients with type 2 diabetes mellitus and atheroma. Arterioscler Thromb Vasc Biol **27**, 525-31 (2007).

14. Wilson PC, Wu H, Kirita Y, Uchimura K, Ledru N, Rennke HG, et al. The single-cell transcriptomic landscape of early human diabetic nephropathy. Proc Natl Acad Sci U S A **116**, 19619-25 (2019).

15. Chella Krishnan K, Vergnes L, Acin-Perez R, Stiles L, Shum M, Ma L, et al. Sex-specific genetic regulation of adipose mitochondria and metabolic syndrome by Ndufv2. Nat Metab **3**, 1552-68 (2021).

16. Chan JY, Bensellam M, Lin RCY, Liang C, Lee K, Jonas JC, et al. Transcriptome analysis of islets from diabetes-resistant and diabetes-prone obese mice reveals novel gene regulatory networks involved in beta-cell compensation and failure. FASEB J **35**, e21608 (2021).

17. Carrero JA, Calderon B, Towfic F, Artyomov MN, Unanue ER. Defining the transcriptional and cellular landscape of type 1 diabetes in the NOD mouse. PLoS One **8**, e59701 (2013).

18. D'Alise AM, Ergun A, Hill JA, Mathis D, Benoist C. A cluster of coregulated genes determines TGF-beta-induced regulatory T-cell (Treg) dysfunction in NOD mice. Proc Natl Acad Sci U S A **108**, 8737-42 (2011).

19. Jain S, Pydi SP, Toti KS, Robaye B, Idzko M, Gavrilova O, et al. Lack of adipocyte purinergic P2Y(6) receptor greatly improves whole body glucose homeostasis. Proc Natl Acad Sci U S A **117**, 30763-74 (2020).

20. Chipitsyna G, Gong Q, Gray CF, Haroon Y, Kamer E, Arafat HA. Induction of monocyte chemoattractant protein-1 expression by angiotensin II in the pancreatic islets and beta-cells. Endocrinology **148**, 2198-208 (2007).

21. Jacobsen PK, Tarnow L, Parving HH. Time to consider ACE insertion/deletion genotypes and individual renoprotective treatment in diabetic nephropathy? Kidney Int **69**, 1293-5 (2006).

22. Zhang X, Shang J, Wang X, Cheng G, Jiang Y, Liu D, et al. Microarray analysis reveals long non-coding RNA SOX2OT as a novel candidate regulator in diabetic nephropathy. Mol Med Rep **18**, 5058-68 (2018).

23. Stechschulte LA, Qiu B, Warrier M, Hinds TD, Jr., Zhang M, Gu H, et al. FKBP51 Null Mice Are Resistant to Diet-Induced Obesity and the PPARgamma Agonist Rosiglitazone. Endocrinology **157**, 3888-900 (2016).

24. Singh M, Kesterson RA, Jacobs MM, Joers JM, Gore JC, Emeson RB. Hyperphagia-mediated obesity in transgenic mice misexpressing the RNA-editing enzyme ADAR2. J Biol Chem **282**, 22448-59 (2007).

25. Pfeifer CW, Walsh JT, Santeford A, Lin JB, Beatty WL, Terao R, et al. Dysregulated CD200-CD200R signaling in early diabetes modulates microglia-mediated retinopathy. Proc Natl Acad Sci U S A **120**, e2308214120 (2023).

26. Miettinen PJ, Ustinov J, Ormio P, Gao R, Palgi J, Hakonen E, et al. Downregulation of EGF receptor signaling in pancreatic islets causes diabetes due to impaired postnatal beta-cell growth. Diabetes **55**, 3299-308 (2006).

27. Kyohara M, Shirakawa J, Okuyama T, Kimura A, Togashi Y, Tajima K, et al. Serum Quantitative Proteomic Analysis Reveals Soluble EGFR To Be a Marker of Insulin Resistance in Male Mice and Humans. Endocrinology **158**, 4152-64 (2017).

28. Zhu Q, Dong H, Bukhari AA, Zhao A, Li M, Sun Y, et al. HUWE1 promotes EGFR ubiquitination and degradation to protect against renal tubulointerstitial fibrosis. FASEB J **34**, 4591-601 (2020).

29. Zhang X, Ohayon-Steckel L, Coppin E, Johny E, Dasari A, Florentin J, et al. Epidermal Growth Factor Receptor in Hepatic Endothelial Cells Suppresses MCP-1-Dependent Monocyte Recruitment in Diabetes. J Immunol **210**, 1363-71 (2023).

30. Cividini F, Scott BT, Suarez J, Casteel DE, Heinz S, Dai A, et al. Ncor2/PPARalpha-Dependent Upregulation of MCUb in the Type 2 Diabetic Heart Impacts Cardiac Metabolic Flexibility and Function. Diabetes **70**, 665-79 (2021).

31. Joshi H, Vastrad B, Joshi N, Vastrad C. Integrated bioinformatics analysis reveals novel key biomarkers in diabetic nephropathy. SAGE Open Med **10**, 20503121221137005 (2022).

32. Ackermann J, Arndt L, Kirstein M, Hobusch C, Brinker G, Kloting N, et al. Myeloid Cell-Specific IL-4 Receptor Knockout Partially Protects from Adipose Tissue Inflammation. J Immunol **207**, 3081-9 (2021).

33. Zaugg J, Melhem H, Huang X, Wegner M, Baumann M, Surbek D, et al. Gestational diabetes mellitus affects placental iron homeostasis: Mechanism and clinical implications. FASEB J **34**, 7311-29 (2020).

34. Muhl L, Mocci G, Pietila R, Liu J, He L, Genove G, et al. A single-cell transcriptomic inventory of murine smooth muscle cells. Dev Cell **57**, 2426-43 e6 (2022).

35. Rondini EA, Granneman JG. Single cell approaches to address adipose tissue stromal cell heterogeneity. Biochem J **477**, 583-600 (2020).

36. Merrick D, Sakers A, Irgebay Z, Okada C, Calvert C, Morley MP, et al. Identification of a mesenchymal progenitor cell hierarchy in adipose tissue. Science **364**, (2019).
