# Supplementary material for: Adipose stem cells are sexually dimorphic cells with dual roles as preadipocytes and resident fibroblasts
Source: Nat Commun. 2024 Sep 2;15:7643. doi: 10.1038/s41467-024-51867-9 (PMC11369120; doi:10.1038/s41467-024-51867-9)
Supplement: Supplementary file 3 — Description of Additional Supplementary Files [file 41467_2024_51867_MOESM3_ESM.pdf]

Description of Additional Supplementary Files

Supplementary Data Legends

Supplementary Data 1: Major scRNAseq papers covering ASCs in white adipose tissue

Supplementary Data 2: In vitro results of adipogenic differentiation from major scRNAseq-ASC papers

Supplementary Movie Legends

Supplementary Movie 1: Movie showing 3D rendering of ASC1 and ASC2 cells in pgWAT in PdgfrbCREdtTOM/PdgfraH2bGFP mice. The staining method and coloring of the different cell types in the videos are: Nuclear PdgfraH2bGFP staining is colored in cyan (showing ASC1 when not co-stained with anti-DPP4 antibody), anti-DPP4 antibody staining for ASC2 cells in green, anti-CD31 antibody staining for endothelial cells in blue and PdgfrbCRE-dtTOM staining for mural cells in red.

Supplementary Movie 2: Movie showing 3D rendering of ASC1 and ASC2 cells in pgWAT in PdgfrbCREdtTOM/PdgfraH2bGFP mice. The staining method and coloring of the different cell types in the videos are: Nuclear PdgfraH2bGFP staining is colored in cyan (showing ASC1 when not co-stained with anti-DPP4 antibody), anti-DPP4 antibody staining for ASC2 cells in green, anti-CD31 antibody staining for endothelial cells in blue and PdgfrbCRE-dtTOM staining for mural cells in red.
