# Supplementary material for: Adipose stem cells are sexually dimorphic cells with dual roles as preadipocytes and resident fibroblasts
Source: Nat Commun. 2024 Sep 2;15:7643. doi: 10.1038/s41467-024-51867-9 (PMC11369120; doi:10.1038/s41467-024-51867-9)
Supplement: Supplementary file 8 — Reporting Summary [file 41467_2024_51867_MOESM8_ESM.pdf]

nature portfolio

Corresponding author(s): Martin Uhrbom

Last updated by author(s): Aug 2, 2024

Reporting Summary

Nature Portfolio wishes to improve the reproducibility of the work that we publish. This form provides structure for consistency and transparency in reporting. For further information on Nature Portfolio policies, see our Editorial Policies and the Editorial Policy Checklist.

Statistics

For all statistical analyses, confirm that the following items are present in the figure legend, table legend, main text, or Methods section.

n/a	Confirmed	
☐	☒	The exact sample size (*n*) for each experimental group/condition, given as a discrete number and unit of measurement
☐	☒	A statement on whether measurements were taken from distinct samples or whether the same sample was measured repeatedly
☐	☒	The statistical test(s) used AND whether they are one- or two-sided<br>*Only common tests should be described solely by name; describe more complex techniques in the Methods section.*
☒	☐	A description of all covariates tested
☐	☒	A description of any assumptions or corrections, such as tests of normality and adjustment for multiple comparisons
☐	☒	A full description of the statistical parameters including central tendency (e.g. means) or other basic estimates (e.g. regression coefficient) AND variation (e.g. standard deviation) or associated estimates of uncertainty (e.g. confidence intervals)
☐	☒	For null hypothesis testing, the test statistic (e.g. *F*, *t*, *r*) with confidence intervals, effect sizes, degrees of freedom and *P* value noted<br>*Give P values as exact values whenever suitable.*
☒	☐	For Bayesian analysis, information on the choice of priors and Markov chain Monte Carlo settings
☒	☐	For hierarchical and complex designs, identification of the appropriate level for tests and full reporting of outcomes
☐	☒	Estimates of effect sizes (e.g. Cohen's *d*, Pearson's *r*), indicating how they were calculated

Our web collection on statistics for biologists contains articles on many of the points above.

Software and code

Policy information about availability of computer code

Data collection

Publicly available dataset were download from to referred web pages in the method section "Other bioinformatic analyses". For comparison to fibroblasts in skeletal muscle and heart, raw fastq-files were provided from Lars Muhl (Reference: 25).

Data analysis

R (4.1.1)
Seurat (4.1.0)
Seurat Object (4.0.4)
tximport (1.22.0)
ggplot (3.3.5)
corrplot (0.92)
DEseq2 (1.30.1)
SingleCellExperiment (1.16.0)
gplots (3.1.1)
Pagoda2(1.0.11)
PrismGraphPad (8.4.3)
FlowJo (10.10)
IncuCyte S3 live-cells analysis system
edgeR (3.22.5)

For manuscripts utilizing custom algorithms or software that are central to the research but not yet described in published literature, software must be made available to editors and reviewers. We strongly encourage code deposition in a community repository (e.g. GitHub). See the Nature Portfolio guidelines for submitting code & software for further information.

Data

Policy information about availability of data

All manuscripts must include a data availability statement. This statement should provide the following information, where applicable:

- Accession codes, unique identifiers, or web links for publicly available datasets
- A description of any restrictions on data availability
- For clinical datasets or third party data, please ensure that the statement adheres to our policy

The RNA-seq raw data generated in this study have been deposited in the NCBI's Gene Expression Omnibus database under accession code GSE273393(scRNA-seq), GSE273413 (FACS_pgWAT_iWAT_ASC), GSE273407 (FACS_castovary_ASC), GSE272408 (bulk_adipocytes) and GSE273416 (in vitro_SVF). The scRNA-seq and bulk RNA-seq data of FACS sorted ASC are available as a searchable database at https://betsholtzlab.org/Publications/WATstromalVascular/database.html.

Research involving human participants, their data, or biological material

Policy information about studies with human participants or human data. See also policy information about sex, gender (identity/presentation), and sexual orientation and race, ethnicity and racism.

Reporting on sex and gender	*Use the terms sex (biological attribute) and gender (shaped by social and cultural circumstances) carefully in order to avoid confusing both terms. Indicate if findings apply to only one sex or gender; describe whether sex and gender were considered in study design; whether sex and/or gender was determined based on self-reporting or assigned and methods used. Provide in the source data disaggregated sex and gender data, where this information has been collected, and if consent has been obtained for sharing of individual-level data; provide overall numbers in this Reporting Summary. Please state if this information has not been collected. Report sex- and gender-based analyses where performed, justify reasons for lack of sex- and gender-based analysis.*
Reporting on race, ethnicity, or other socially relevant groupings	*Please specify the socially constructed or socially relevant categorization variable(s) used in your manuscript and explain why they were used. Please note that such variables should not be used as proxies for other socially constructed/relevant variables (for example, race or ethnicity should not be used as a proxy for socioeconomic status). Provide clear definitions of the relevant terms used, how they were provided (by the participants/respondents, the researchers, or third parties), and the method(s) used to classify people into the different categories (e.g. self-report, census or administrative data, social media data, etc.) Please provide details about how you controlled for confounding variables in your analyses.*
Population characteristics	*Describe the covariate-relevant population characteristics of the human research participants (e.g. age, genotypic information, past and current diagnosis and treatment categories). If you filled out the behavioural & social sciences study design questions and have nothing to add here, write "See above."*
Recruitment	*Describe how participants were recruited. Outline any potential self-selection bias or other biases that may be present and how these are likely to impact results.*
Ethics oversight	*Identify the organization(s) that approved the study protocol.*

Note that full information on the approval of the study protocol must also be provided in the manuscript.

Field-specific reporting

Please select the one below that is the best fit for your research. If you are not sure, read the appropriate sections before making your selection.

☒ Life sciences ☐ Behavioural & social sciences ☐ Ecological, evolutionary & environmental sciences

For a reference copy of the document with all sections, see nature.com/documents/nr-reporting-summary-flat.pdf

Life sciences study design

All studies must disclose on these points even when the disclosure is negative.

Sample size	The sample size for scRNAseq experiments was chosen so that at least cells from three mice from each sex were collected. The sample size for FACS sorted bulk-RNA sequencing was chosen to eight mice in each group, this group size was determined based on purpose of maintaining good/efficient sample collection and providing enough biological replicates for statistically assessing the differentially expressed genes between the groups. The rest of the experiments had 1-4 mice and were repeated at least three times, no sample size calculations were performed.
Data exclusions	Exclusion of scRNAseq and bulk-RNA seq samples were based on the QC-filtering as described in the method section. For in vitro differentiated FACS sorted ASCs, wells with cell viability below 85% were not included in the analysis.
Replication	In vitro experiments were replicated at least three times, independently and under identical conditions. One exception is the bulk RNA sequencing samples of in vitro cultured SVF cells from perigonadal white adipose tissue. These samples are from two independent experiments with a minimum of two technical replicates each.

	Findings from the scRNAseq data regarding sexual dimorphic genes in ASC were validated with Bulk-RNAseq samples from FACS-sorted ASC and publicly available scRNAseq datasets.
Randomization	For animal experiments, mice were group according to sex.<br>For cell experiments in vitro, samples were group according to sex and fat depot. Groups were distributed symmetrically across the plate without using the wells along the edges of the plate, these well were filled with PBS instead of medium to minimize edge effects.<br>The order for which samples were collected and analyzed were randomized for bulk RNA-seq samples of FACS sorted ASC.
Blinding	For in vivo experiments, investigators were not blinded since the mice were group according to sex and this trait will be apparent for the investigator even if it was blinded with anonymous mouse ID.

Reporting for specific materials, systems and methods

We require information from authors about some types of materials, experimental systems and methods used in many studies. Here, indicate whether each material, system or method listed is relevant to your study. If you are not sure if a list item applies to your research, read the appropriate section before selecting a response.

Materials & experimental systems

n/a	Involved in the study
☐	☒ Antibodies
☐	☐ Eukaryotic cell lines
☐	☐ Palaeontology and archaeology
☐	☒ Animals and other organisms
☐	☐ Clinical data
☐	☐ Dual use research of concern
☐	☐ Plants

Methods

n/a	Involved in the study
☐	☐ ChIP-seq
☐	☒ Flow cytometry
☐	☐ MRI-based neuroimaging

Antibodies

Antibodies used	FACS: BD Horizon BV421 Rat Anti-Mouse CD31, cat#:562939, Clone MEC 13.3, BD Bioscience<br>FACS: BD Pharmingen™ APC-Cy™7 Rat Anti-Mouse CD45, cat#:557659, Clone 30-F11, BD Bioscience<br>FACS: BD Pharmingen™ FITC Rat anti-Mouse CD34,c at#:553733, Clone RAM34, BD Bioscience<br>FACS: PE anti-mouse CD26 (DPP-4) Antibody, cat#:137804, Clone H194-112, Biolegend<br>IHC: Human/Mouse/Rat CD31/PECAM-1 Antibody, cat#:AF3628, Polyclonal Goat IgG,lot:YZU0114021 / YZU0121071, R&D.<br>IHC: BD Pharmingen™ Purified Rat Anti-Mouse CD31,cat#:550274,MEC13.3, lot:53198, BD Bioscience.<br>IHC: Anti-CD31 antibody, cat#:ab28364,Rabbit Polyclonal ,lot:GR3247742-11, Abcam.<br>IHC: Mouse DPPIV/CD26 Antibody, cat#:AF954, Polyclonal Goat IgG, lot:GJT0318011, R&D.<br>IHC: Mouse PDGF R alpha Antibody, cat#:AF1062, Polyclonal Goat IgG, lot:HMQ0216021,R&D.<br>IHC: Anti-p75 NGF Receptor antibody, cat#:ab5287,Monoclonal Rabbit IgG, lot:GR3238403-3<br>Secondary for IHC:<br>Donkey anti-Goat IgG (H+L) Cross-Adsorbed Secondary Antibody, Alexa Fluor 633,cat#:A21082, Host:Donkey, lot:1889311 / 2309146, company:Invitrogen<br>Donkey anti-Goat IgG (H+L) Cross-Adsorbed Secondary Antibody, Alexa Fluor 555, cat#:A21432, Host:Donkey, lot:1818686, company:Invitrogen<br>Donkey anti-Rabbit IgG (H+L) Highly Cross-Adsorbed Secondary Antibody, Alexa Fluor 680,cat#:A10043,Host:Donkey, lot:1917929 / 2165747, company:Invitrogen<br>Donkey anti-Rabbit IgG (H+L) Highly Cross-Adsorbed Secondary Antibody, Alexa Fluor 555, cat#:A31572,Host:Donkey, lot:1837922, company:Invitrogen<br>Donkey anti-Rat IgG (H+L) Highly Cross-Adsorbed Secondary Antibody, Alexa Fluor 488, cat#:A21208,Host:Donkey, lot:1900239, company:Invitrogen<br>Donkey anti-Rat IgG (H+L) Cross-Adsorbed Secondary Antibody, DyLight 650, cat#:SA5-10029, Host:Donkey, lot:RK2304679, company:Invitrogen<br>Donkey anti-Rat IgG (H+L) Cross-Adsorbed Secondary Antibody, DyLight 680, cat#:SA5-10030, Host:Donkey, lot:VC2968071, company:Invitrogen<br>Cy3 AffiniPure™ Donkey Anti-Rat IgG (H+L)m, cat#: 712-165-153,Host:Donkey, lot:139289, company:Jackson Laboratories.
Validation	he antibodies used for flow cytometry are against well established targets and have been QC-tested by the suppliers.<br>Human/Mouse/Rat CD31/PECAM-1 Antibody,cat#:AF3628: Specificity statement on webpage: Detects human and mouse CD31/ PECAM-1 in direct ELISAs and Western blots. In direct ELISAs and Western blots, approximately 10% cross-reactivity with recombinant porcine CD31 is observed.<br>Anti-CD31 antibody, ab28364, Abcam: webpage statement: Validated in IHC-P and tested in Human samples.<br>Mouse DPPIV/CD26 Antibody, AF954, Specificity statement on webpage: Detects mouse DPPIV/CD26 in direct ELISAs and Western blots. In direct ELISAs, less than 20% cross reactivity with recombinant human DPPIV/CD26 is observed<br>Mouse PDGFR alpha Antibody, cat#:AF1062: Specificity statement on webpage:Detects mouse PDGF R alpha in direct ELISAs and Western blots. In direct ELISAs, less than 1% cross-reactivity with recombinant human (rh) PDGF R alpha, rhPDGF R beta, and recombinant mouse PDGFR beta is observed.<br>Anti-p75 NGF Receptor antibody, cat#:ab5287, webpage statement:Validated in IP, WB, ICC/IF, Flow Cyt (Intra), IHC-P and tested in Rat, Mouse, Human samples.<br>For secondary:

Cy3 AffiniPure Donkey Anti-Rat IgG (H+L)m, cat#: 712-165-153,Host:Donkey, lot:139289, company:Jackson Laboratories. statement on webpage: Based on immunoelectrophoresis and/or ELISA, the antibody reacts with whole molecule rat IgG. It also reacts with the light chains of other rat immunoglobulins. No antibody was detected against non-immunoglobulin serum proteins. The antibody has been tested by ELISA and/or solid-phase adsorbed to ensure minimal cross-reaction with bovine, chicken, goat, guinea pig, syrian hamster, horse, human, mouse, rabbit and sheep serum proteins, but it may cross-react with immunoglobulins from other species. The rest of the secondary antibodies are from Invitrogen, antibody testing data that validates the selectivity of the antibodies are provided on their webpage.

Eukaryotic cell lines

Policy information about cell lines and Sex and Gender in Research

Cell line source(s)

State the source of each cell line used and the sex of all primary cell lines and cells derived from human participants or vertebrate models.

Authentication

Describe the authentication procedures for each cell line used OR declare that none of the cell lines used were authenticated.

Mycoplasma contamination

Confirm that all cell lines tested negative for mycoplasma contamination OR describe the results of the testing for mycoplasma contamination OR declare that the cell lines were not tested for mycoplasma contamination.

Commonly misidentified lines (See ICLAC register)

Name any commonly misidentified cell lines used in the study and provide a rationale for their use.

Palaeontology and Archaeology

Specimen provenance

Provide provenance information for specimens and describe permits that were obtained for the work (including the name of the issuing authority, the date of issue, and any identifying information). Permits should encompass collection and, where applicable, export.

Specimen deposition

Indicate where the specimens have been deposited to permit free access by other researchers.

Dating methods

If new dates are provided, describe how they were obtained (e.g. collection, storage, sample pretreatment and measurement), where they were obtained (i.e. lab name), the calibration program and the protocol for quality assurance OR state that no new dates are provided.

☐ Tick this box to confirm that the raw and calibrated dates are available in the paper or in Supplementary Information.

Ethics oversight

Identify the organization(s) that approved or provided guidance on the study protocol, OR state that no ethical approval or guidance was required and explain why not.

Note that full information on the approval of the study protocol must also be provided in the manuscript.

Animals and other research organisms

Policy information about studies involving animals; ARRIVE guidelines recommended for reporting animal research, and Sex and Gender in Research

Laboratory animals

For scRNAseq: PdgfrbGFP (Genesat.org, Tg(Pdgfrb-eGFP)) mouse strain that have been backcrossed to the C57BL6/J background (The Jackson Laboratory, C57B16/J), Age: 12-20 weeks.

For Imaging: PdgfrbGFP (Genesat.org, Tg(Pdgfrb-eGFP)) mouse strain that have been backcrossed to the C57BL6/J background (The Jackson Laboratory, C57B16/J) for images in figure 5.
PdgfraH2b-GFP (B6.Cg-Pdgfratm11(EGFP)Sor) mice were crossed to Pdgfrb-CreERT2 (Tg(Pdgfrb-CRE/ERT2)6096Rha) and Ail4-TdTomato(B6.Cg-Gt(ROSA)26Sortm14 (CAG-TdTomato)Hze) for images in figure 6 and videos. Age: 12-20 weeks.

For FACS sorted ASC bulk-RNAseq: C57BL/6J mouse strain (supplied by Charles River), Age at termination: 18 weeks
For the castration/ovariectomy study, ovariectomized (study code: OVARIEX), castrated (Study code: CASTRATE) and aged matched controls of the strain C57BL/6J were supplied from Charles River and terminated at 10 weeks of age.

For in vitro experiments in figure 4e-f, wild-type C57BL/6N mouse strain were used and for the rest of the in vitro experiments we used C57BL/6J mouse strain both strains were supplied by Charles River. Age: 12-20 weeks.

Wild animals

No wild animals were used.

Reporting on sex

Groups of male and female mice were used throughout the work and the differences between the sexes were compared for the majority of the experiments. Results in Figure 4b, showing proliferation rate of ASC was based on only female mice, similar results is expected in male mice since this data has been published previously. No comparison between male and female mice have been done for the proliferation rate since indications that it would be a difference are lacking, the ASC groups in the experiments assessing differentiation capacity (Fig 4e) resulted in similar level of confluency after 3-4 days of proliferation when seeded at the same cell density. For the gene expression measurements in Fig 4d, both male and female mice were used however the results are pooled since the size of the groups are to small to assess any difference between the sexes.

Field-collected samples	*For laboratory work with field-collected samples, describe all relevant parameters such as housing, maintenance, temperature, photoperiod and end-of-experiment protocol OR state that the study did not involve samples collected from the field.*
Ethics oversight	All mouse experiments were conducted according to local guidelines and regulations for animal welfare, experiments on reporter mice strains were covered by ethical permits approved by Linköping's animal Research Ethics, approval ID 729 and 3711-2020, whereas experiments with wild-type mice for in vitro studies and FACS bulk RNA-seq isolations were covered by ethical permits approved by Gothenburg's animal research Ethics committee, approval ID: 000832-2017.

Note that full information on the approval of the study protocol must also be provided in the manuscript.

Clinical data

Policy information about clinical studies

All manuscripts should comply with the ICMJE guidelines for publication of clinical research and a completed CONSORT checklist must be included with all submissions.

Clinical trial registration	*Provide the trial registration number from ClinicalTrials.gov or an equivalent agency.*
Study protocol	*Note where the full trial protocol can be accessed OR if not available, explain why.*
Data collection	*Describe the settings and locales of data collection, noting the time periods of recruitment and data collection.*
Outcomes	*Describe how you pre-defined primary and secondary outcome measures and how you assessed these measures.*

Dual use research of concern

Policy information about dual use research of concern

Hazards

Could the accidental, deliberate or reckless misuse of agents or technologies generated in the work, or the application of information presented in the manuscript, pose a threat to:

No	Yes	
☐	☐	Public health
☐	☐	National security
☐	☐	Crops and/or livestock
☐	☐	Ecosystems
☐	☐	Any other significant area

Experiments of concern

Does the work involve any of these experiments of concern:

No	Yes	
☐	☐	Demonstrate how to render a vaccine ineffective
☐	☐	Confer resistance to therapeutically useful antibiotics or antiviral agents
☐	☐	Enhance the virulence of a pathogen or render a nonpathogen virulent
☐	☐	Increase transmissibility of a pathogen
☐	☐	Alter the host range of a pathogen
☐	☐	Enable evasion of diagnostic/detection modalities
☐	☐	Enable the weaponization of a biological agent or toxin
☐	☐	Any other potentially harmful combination of experiments and agents

Plants

Seed stocks | *Report on the source of all seed stocks or other plant material used. If applicable, state the seed stock centre and catalogue number. If plant specimens were collected from the field, describe the collection location, date and sampling procedures.*

Novel plant genotypes | *Describe the methods by which all novel plant genotypes were produced. This includes those generated by transgenic approaches, gene editing, chemical/radiation-based mutagenesis and hybridization. For transgenic lines, describe the transformation method, the number of independent lines analyzed and the generation upon which experiments were performed. For gene-edited lines, describe the editor used, the endogenous sequence targeted for editing, the targeting guide RNA sequence (if applicable) and how the editor was applied.*

Authentication | *Describe any authentication procedures for each seed stock used or novel genotype generated. Describe any experiments used to assess the effect of a mutation and, where applicable, how potential secondary effects (e.g. second site T-DNA insertions, mosiacism, off-target gene editing) were examined.*

ChIP-seq

Data deposition

☐ Confirm that both raw and final processed data have been deposited in a public database such as GEO.

☐ Confirm that you have deposited or provided access to graph files (e.g. BED files) for the called peaks.

Data access links
May remain private before publication. | *For "Initial submission" or "Revised version" documents, provide reviewer access links. For your "Final submission" document, provide a link to the deposited data.*

Files in database submission | *Provide a list of all files available in the database submission.*

Genome browser session
(e.g. UCSC) | *Provide a link to an anonymized genome browser session for "Initial submission" and "Revised version" documents only, to enable peer review. Write "no longer applicable" for "Final submission" documents.*

Methodology

Replicates | *Describe the experimental replicates, specifying number, type and replicate agreement.*

Sequencing depth | *Describe the sequencing depth for each experiment, providing the total number of reads, uniquely mapped reads, length of reads and whether they were paired- or single-end.*

Antibodies | *Describe the antibodies used for the ChIP-seq experiments; as applicable, provide supplier name, catalog number, clone name, and lot number.*

Peak calling parameters | *Specify the command line program and parameters used for read mapping and peak calling, including the ChIP, control and index files used.*

Data quality | *Describe the methods used to ensure data quality in full detail, including how many peaks are at FDR 5% and above 5-fold enrichment.*

Software | *Describe the software used to collect and analyze the ChIP-seq data. For custom code that has been deposited into a community repository, provide accession details.*

Flow Cytometry

Plots

Confirm that:

☒ The axis labels state the marker and fluorochrome used (e.g. CD4-FITC).

☒ The axis scales are clearly visible. Include numbers along axes only for bottom left plot of group (a 'group' is an analysis of identical markers).

☒ All plots are contour plots with outliers or pseudocolor plots.

☒ A numerical value for number of cells or percentage (with statistics) is provided.

Methodology

Sample preparation | Mice were euthanized according to the ethical permission by cervical dislocation before inguinal/perigonadal white adipose tissue was removed and placed into cold PBS solution. The adipose tissue was then cut into smaller pieces before incubation in dissociation buffer (Skeletal Muscle dissociation kit, Miltenyi), supplemented with 1 mg/ml Collagenase type IV-S at 37 °C with horizontal shaking at 500-800 rpm. For all in vitro experiments a different enzymatic mixture was used with 2 mg/ml Dispase ii, 1 mg/ml Collagenase I, 1 mg/ml Collagenase II and 25 units/ml of DNAse dissolved in DMEM. The tissue was further disintegrated by pipetting every 10 minutes during the 30-minute-long incubation. The cell suspension was then sequentially passed through a 70 µm and 40 µm cell strainers, before 5 ml of DMEM was passed through both strainers as

	final washing step. Cells were then spun at 300xg for 5 min, the buffer was removed, and the pellet was re-suspended in FACS buffer (PBS, supplemented with 0.5% BSA, 2 mM EDTA, 25 mM HEPES). Cells were then labeled with fluorophore-conjugated antibodies (anti-CD31, anti-CD34, anti-DPP4, anti-CD45) for 20 min on ice, then centrifugated at 250 g for 5 min, after removal of the supernatant the pellet was re-suspended with FACS buffer and kept on ice.
Instrument	Beckson Dickson FACS Aria III or FACS Melody Cells instruments equipped with 100 µm nozzle were used for sorting cells into individual-wells for scRNAseq experiments.<br>SH800 Sony cell sorter(100 um nozzle chip) and FACS Aria III was used for in vitro experiments and bulk-RNAseq of ASCs.
Software	For the FACS Aria III-instrument BD FACS Diva software version 9.4 was used.<br>For the SH800 Sony cell sorter instrument "Cell sorter software" version 2.1.6 was used.<br>FlowJo version 10.10 was used to analyze the data.
Cell population abundance	The purity of the samples was determine by analyzing the gene expression of bulk-RNAseq samples of the FACS sorted subpopulations of ASC, as shown in supplementary figure 3-4.
Gating strategy	For FACS-sorting of single cells: Cell suspension derived from Pdgfrb-eGFP reporter mice were stained with antibodies and subjected to flow cytometry sorting as described previously (ref 25). First, a gate of forward and side scatter area (FSC-A/SSC-A) on the linear scale was set generously in order to only eliminate cells with low values (red blood cells and cell debris), a second gate for double discrimination was used based on distance from the diagonal line in the FSC-A/FSC-height plot, the third selection criteria was based on fluorescent signaling, with “fluorescent minus one” or mice negative for the GFP-reporter used as gating controls. Cells negative for CD45-staining were first selected, further gating were then either based on PdgfrbGFP-/CD31+, CD31-/ PdgfrbGFP+ or CD31-/ PdgfrbGFP+/ DPP4± selections.<br>For FACS sorting for in vitro experiments and bulk-RNAseq of ASCs:<br>Stromal vascular cells from both iWAT and pgWAT were isolated according to the procedure described above. Thereafter cells were labelled with four fluorophore-conjugated antibodies (anti-CD45, anti-CD31, anti-CD34 and anti-DPP4) for 30 minutes on ice. The cell suspension was then centrifuged at 300 g for 5 min, supernatant removed, and pellet re-suspended in FACS-buffer. Cells were then loaded into a SH800 Sony cell sorter, and two adipose stem cell populations, CD45-/CD31-/CD34+/DPP4+ and CD45-/CD31-/CD34+/DPP4-, were gated and selected for sorting. Fluorescent minus one controls were used for ensuring correct gating as seen in supplementary figure 3.

☒ Tick this box to confirm that a figure exemplifying the gating strategy is provided in the Supplementary Information.

Magnetic resonance imaging

Experimental design

Design type	*Indicate task or resting state; event-related or block design.*
Design specifications	*Specify the number of blocks, trials or experimental units per session and/or subject, and specify the length of each trial or block (if trials are blocked) and interval between trials.*
Behavioral performance measures	*State number and/or type of variables recorded (e.g. correct button press, response time) and what statistics were used to establish that the subjects were performing the task as expected (e.g. mean, range, and/or standard deviation across subjects).*

Acquisition

Imaging type(s)	*Specify: functional, structural, diffusion, perfusion.*
Field strength	*Specify in Tesla*
Sequence & imaging parameters	*Specify the pulse sequence type (gradient echo, spin echo, etc.), imaging type (EPI, spiral, etc.), field of view, matrix size, slice thickness, orientation and TE/TR/flip angle.*
Area of acquisition	*State whether a whole brain scan was used OR define the area of acquisition, describing how the region was determined.*

Diffusion MRI ☐ Used ☐ Not used

Preprocessing

Preprocessing software	*Provide detail on software version and revision number and on specific parameters (model/functions, brain extraction, segmentation, smoothing kernel size, etc.).*
Normalization	*If data were normalized/standardized, describe the approach(es): specify linear or non-linear and define image types used for transformation OR indicate that data were not normalized and explain rationale for lack of normalization.*
Normalization template	*Describe the template used for normalization/transformation, specifying subject space or group standardized space (e.g. original Talairach, MNI305, ICBM152) OR indicate that the data were not normalized.*
Noise and artifact removal	*Describe your procedure(s) for artifact and structured noise removal, specifying motion parameters, tissue signals and physiological signals (heart rate, respiration).*

Volume censoring | *Define your software and/or method and criteria for volume censoring, and state the extent of such censoring.*

Statistical modeling & inference

Model type and settings | *Specify type (mass univariate, multivariate, RSA, predictive, etc.) and describe essential details of the model at the first and second levels (e.g. fixed, random or mixed effects; drift or auto-correlation).*

Effect(s) tested | *Define precise effect in terms of the task or stimulus conditions instead of psychological concepts and indicate whether ANOVA or factorial designs were used.*

Specify type of analysis: ☐ Whole brain ☐ ROI-based ☐ Both

Statistic type for inference | *Specify voxel-wise or cluster-wise and report all relevant parameters for cluster-wise methods.*

(See Eklund et al. 2016)

Correction | *Describe the type of correction and how it is obtained for multiple comparisons (e.g. FWE, FDR, permutation or Monte Carlo).*

Models & analysis

n/a	Involved in the study
☐	☐ Functional and/or effective connectivity
☐	☐ Graph analysis
☐	☐ Multivariate modeling or predictive analysis

Functional and/or effective connectivity | *Report the measures of dependence used and the model details (e.g. Pearson correlation, partial correlation, mutual information).*

Graph analysis | *Report the dependent variable and connectivity measure, specifying weighted graph or binarized graph, subject- or group-level, and the global and/or node summaries used (e.g. clustering coefficient, efficiency, etc.).*

Multivariate modeling and predictive analysis | *Specify independent variables, features extraction and dimension reduction, model, training and evaluation metrics.*
